# Unique microbial landscape in the human oropharynx during different types of acute respiratory tract infections

**DOI:** 10.1186/s40168-023-01597-9

**Published:** 2023-07-24

**Authors:** Hui Li, Xiaorong Wu, Hong Zeng, Bozhen Chang, Ying Cui, Jingxiang Zhang, Ruixia Wang, Tao Ding

**Affiliations:** 1 Department of Immunology and Microbiology, Zhongshan School of Medicine, Sun Yat-Sen University, Guangzhou, 510080 China; 2 Key Laboratory of Tropical Diseases Control (Sun Yat-Sen University), Ministry of Education, Guangzhou, 510080 China; 3 Center for Disease Control and Prevention of Nanhai District, Foshan, 528200 China

**Keywords:** Influenza A virus, Microbiome, Upper respiratory tract, Pathogen

## Abstract

**Background:**

Secondary bacterial infections and pneumonia are major mortality causes of respiratory viruses, and the disruption of the upper respiratory tract (URT) microbiota is a crucial component of this process. However, whether this URT dysbiosis associates with the viral species (in other words, is viral type-specific) is unclear.

**Results:**

Here, we recruited 735 outpatients with upper respiratory symptoms, identified the infectious virus types in 349 participants using multiplex RT-PCR, and profiled their upper respiratory microbiome using the 16S ribosomal RNA gene and metagenomic gene sequencing. Microbial and viral data were subsequently used as inputs for multivariate analysis aimed at revealing viral type-specific disruption of the upper respiratory microbiota. We found that the oropharyngeal microbiota shaped by influenza A virus (FluA), influenza B virus (FluB), respiratory syncytial virus (RSV), and human rhinovirus (HRV) infections exhibited three distinct patterns of dysbiosis, and *Veillonella* was identified as a prominent biomarker for any type of respiratory viral infections. Influenza virus infections are significantly correlated with increased oropharynx microbiota diversity and enrichment of functional metabolic pathways such as L-arginine biosynthesis and tetracycline resistance gene *tetW*. We used the GRiD algorithm and found the predicted growth rate of common respiratory pathogens was increased upon influenza virus infection, while commensal bacteria, such as *Streptococcus infantis* and *Streptococcus mitis*, may act as a colonization resistance to the overgrowth of these pathogens.

**Conclusions:**

We found that respiratory viral infections are linked with viral type-specific disruption of the upper respiratory microbiota, particularly, influenza infections uniquely associated with increased microbial diversity and growth rates of specific pathogens in URT. These findings are essential for clarifying the differences and dynamics of respiratory microbiota in healthy participants and acute respiratory viral infections, which contribute to elucidating the pathogenesis of viral-host-bacterial interactions to provide insights into future studies on effective prevention and treatment of respiratory tract infections.

Video Abstract

**Supplementary Information:**

The online version contains supplementary material available at 10.1186/s40168-023-01597-9.

## Introduction

Respiratory viruses, such as influenza (Flu) and human respiratory syncytial virus (RSV), are major threats to global public health [1]. Respiratory viral infections predispose patients to secondary bacterial infections and are the leading cause of death from infectious diseases [2–4]. It is crucial to study the major causes of bacterial infections and the factors that influence symptoms or outcomes; however, the interplay of multiple factors, such as viruses, bacteria, and the host immune system, complicates the elucidation of bacterial infections [5, 6]. Current advances in microbiome research have led to a significant change in our view of bacteria: more than simply pathogens, they are a community that can live in symbiosis with the human body [7]. Microbes form a complex system (i.e., the microbiome) closely involved in human health and various conditions [8]. Viral infections disrupt the microbial-host balance maintained by this complex system [9]. Yet, this process is so complicated due to the factors involved that we are still unclear about its exact course and whether it is viral type-specific.

The respiratory microbiome is considered the gatekeeper of the respiratory tract, although its clinical implications in respiratory diseases remain largely unknown [10]. The respiratory tract can be divided into two parts, upper and lower respiratory tracts (URT and LRT, respectively), and the former has a significantly higher microbial density than the latter. In URT, the oropharynx is an important site of infection and replication for most respiratory viruses [11]. Meanwhile, the oropharyngeal microbiota is considered a major source of lung microbiota, even in healthy adults, and is involved in secondary pneumonia [10, 12]. Among viral infections, influenza virus and RSV have been well studied for their epidemics and high mortality caused by bacterial pneumonia [13–15]. For example, the results from a meta-analysis show that the proportion of secondary bacterial infections following influenza infection is up to 65% [16], with the majority ranging from 11.5 to 34% [14, 17–19]. The secondary bacterial infections in RSV infection is up to 32.4% [20–22], and human rhinovirus (HRV) infection ranges from 9.1 to 18.9% [23, 24]. At the same time, in case reports of influenza infection, patients with higher influenza-mediated secondary infections also have a higher disease burden [17], and nearly 30% of severe influenza patients are exacerbated by co-infection with *Streptococcus pneumoniae*, *Staphylococcus aureus*, and *Haemophilus influenzae* in the lung [25, 26]. With the development of microbiome studies, the similarities and differences of dysbacteriosis characteristics upon different viral infections are anticipated to explore and further characterize the intrinsic connection between this dysbiosis and secondary bacterial infections of the lower respiratory tract.

Several studies based on 16S rRNA gene and metagenomic sequencing have reported the disruption of the respiratory microbiota with influenza infection [27–30]; however, it remains unclear whether this disruption is viral type-specific due to the complexity of clinical viral infections, such as mixed infections with various viruses. This hinders further studies on how viruses act in an inflammatory environment and then influence the respiratory microbiota, contributing to a different outcome of bacterial infections in the respiratory tract. Therefore, human studies based on large sample sizes of multiple viral infections are needed to reveal the role and the mechanism of viral infections in shaping the respiratory microbiome. In the present study, we identified 349 patients with single or multiple infections of FluA, FluB, RSV, and human rhinovirus (HRV) from 735 patients using a multiplex RT-PCR approach from March to August 2019 and recruited 98 healthy participants as the control group. We collected oropharyngeal swabs (representing URT specimens) and performed 16S rRNA gene and metagenomic sequencing to profile the microbiome and elucidate the respiratory dysbiosis.

## Methods

### Study participants and sample preparation

This study included 735 influenza-like cases from the outpatient and 98 healthy participants from March to August 2019 at Nanhai Hospital of Southern Medical University (Foshan, Guangdong, China). Patients with three or more clinical symptoms, including fever, cough, sore throat, runny or stuffy nose, body aches or headaches, and fatigue, would be involved in this study. All participants provided written informed consent. The demographic characteristics including age, gender, height, weight, influenza vaccination records, and chronic respiratory diseases were recorded (Table 1).

**Table 1:** Demographics and infection characteristics of the participants included in this study

Level	Overall	Health^a^	FluA	FluB	HRV	RSV	FluA/FluB	FluA/FluB/HRV	FluA/FluB/RSV	FluA/HRV	FluA/RSV	FluB/HRV	FluB/RSV	FluB/HRV/RSV	HRV/RSV	FluA/FluB/HRV/RSV
***n***	447	98	22	138	61	34	16	4	12	3	13	22	16	3	3	2
**BMI** (*Significant *P.adj*)			*ns*	*ns*	*ns*	*ns*	*ns*	*ns*	*ns*	*ns*	*ns*	*ns*	*ns*	*ns*	*ns*	*ns*
Median [IQR], kg/m^2^	21.22 [19.04, 23.88]	20.50 [18.72, 22.58]	22.34 [19.38, 23.80]	21.48 [19.22, 24.49]	20.31 [19.13, 23.48]	22.60 [18.73, 26.12]	20.76 [19.76, 21.34]	22.50 [21.96, 29.50]	26.37 [19.16, 29.42]	19.13 [18.93, 23.34]	20.74 [18.79, 24.34]	21.10 [18.94, 22.70]	21.16 [19.33, 24.08]	20.72 [20.36, 31.38]	23.44 [20.91, 32.35]	22.19 [21.32, 23.07]
**Sex** (*Significant *P.adj*)			*	*	*	*	*	*	*	*	*ns*	*	*	*ns*	*ns*	*ns*
Female, *n* (%)	264 (59.1)	88 (89.8)	11 (50.0)	72 (52.2)	30 (49.2)	19 (55.9)	5 (31.2)	1 (25.0)	6 (50.0)	1 (33.3)	9 (69.2)	10 (45.5)	7 (43.8)	2 (66.7)	2 (66.7)	1 (50.0)
Male, *n* (%)	178 (39.8)	8 ( 8.2)	11 (50.0)	66 (47.8)	30 (49.2)	14 (41.2)	11 (68.8)	2 (50.0)	6 (50.0)	2 (66.7)	4 (30.8)	12 (54.5)	9 (56.2)	1 (33.3)	1 (33.3)	1 (50.0)
**Age**^b^ (*Significant *P.adj*)			*ns*	*ns*	*ns*	*ns*	*ns*	*ns*	*ns*	*ns*	*ns*	*ns*	*ns*	*ns*	*ns*	*ns*
Median [IQR], years	29.00 [23.50, 35.00]	31.50 [24.75, 38.00]	29.00 [27.00, 37.50]	29.00 [24.00, 35.00]	26.00 [22.00, 32.00]	30.00 [25.00, 36.00]	29.00 [26.50, 31.00]	30.50 [28.25, 32.75]	29.00 [27.00, 32.25]	43.00 [37.50, 56.50]	34.00 [28.00, 55.00]	26.50 [22.50, 30.00]	21.00 [19.00, 25.75]	23.00 [20.50, 26.00]	47.00 [36.50, 54.50]	23.50 [20.25, 26.75]
**Vaccine** (*Significant *P.adj*)			*ns*	*ns*	*ns*	*ns*	*ns*	*ns*	*ns*	*ns*	*	*ns*	*ns*	*ns*	*	*ns*
No, *n* (%)	347 (77.6)	74 (75.5)	15 (68.2)	106 (76.8)	50 (82.0)	29 (85.3)	12 (75.0)	4 (100.0)	10 (83.3)	2 (66.7)	9 (69.2)	20 (90.9)	11 (68.8)	1 (33.3)	2 (66.7)	2 (100.0)
Yes, *n* (%)	80 (17.9)	24 (24.5)	6 (27.3)	24 (17.4)	7 (11.5)	5 (14.7)	3 (18.8)	0 ( 0.0)	1 ( 8.3)	1 (33.3)	1 ( 7.7)	1 ( 4.5)	5 (31.2)	2 (66.7)	0 ( 0.0)	0 ( 0.0)
**Chronic respiratory diseases** (*Significant *P.adj*)			*ns*	*ns*	*ns*	*ns*	*ns*	*ns*	*ns*	*ns*	*	*ns*	*ns*	*ns*	*ns*	*ns*
No, *n* (%)	345 (77.2)	86 (87.8)	18 (81.8)	101 (73.2)	44 (72.1)	27 (79.4)	12 (75.0)	3 (75.0)	9 (75.0)	1 (33.3)	10 (76.9)	16 (72.7)	12 (75.0)	2 (66.7)	2 (66.7)	2 (100.0)
Yes, *n* (%)	93 (20.8)	12 (12.2)	4 (18.2)	33 (23.9)	16 (26.2)	7 (20.6)	3 (18.8)	1 (25.0)	3 (25.0)	2 (66.7)	1 ( 7.7)	5 (22.7)	4 (25.0)	1 (33.3)	1 (33.3)	0 ( 0.0)

Continuous Variables are presented as median (interquartile range) unless otherwise indicated

Chronic respiratory diseases: lung cancer, chronic obstructive pneumonia disease, bronchitis, idiopathic pulmonary fibrosis

*IQR* Interquartile range, *BMI* Body mass index, *FluA* Influenza A virus, *FluB* Influenza B virus, *RSV* Respiratory syncytial virus, *HRV* Human rhinovirus

^*^Significant *P.adjust* (Bonferroni *P.adjust* < 0.05) were calculated by using the Mann-Whitney *U* test or the Fisher exact test between subjects with one specific virus to the healthy controls; *ns*, non-significant

^a^Healthy controls of this study were recruited from paramedics at the same community hospitals in this study

^b^Age stratification of this cohort included adolescents (13-18 years, *n* = 53) and adults (19 years and older, *n* = 372)

Two oropharyngeal samples were obtained from each patient for further testing: one with a FLOQSwabs (Copan, FLOQSwabs™) for RNA extraction was diluted in 3 mL of Universal Transport Medium™ (Copan, UTM™), and the other with a sterile swab (Copan, 108CSR) for DNA extraction was flashed frozen with dry ice and transferred within 1 h to a-80 °C refrigerator. The FLOQSwabs™ swab was collected first, followed by sterile swabs. Each swab was used to wipe the root of the tongue and the pharyngeal tonsils and the posterior pharyngeal wall six times on both sides of the subject.

### RNA extraction, multiplex RT-PCR, and laboratory diagnosis

Total RNA was extracted with RNAiso Plus (Takara, 9109) and then detected by multiplex RT-PCR with a PrimeScript^TM^RT Master Mix (Takara, RR036A) reverse transcription kit using primers and probes (Additional file 1: Table S1) targeting FluA, FluB, HRV, and RSV as previously [31]. Synthetic plasmids targeting FluA, FluB, HRV, and RSV were used to produce standard curves (Additional file 2: Fig. S1). The multiplex RT-PCR cycles were initiated at 95 °C for 5 min, followed by 40 cycles at 95 °C for 5 s, 60 °C for 30 s. Three replicate wells were set up for each sample amplification, and specimens were considered positive when the Ct value of one well was less than 37 or two wells were between 37 and 39. Among 735 influenza-like cases, 349 samples were identified as single positive or multiple positive (two or more viruses) for FluA, FluB, HRV, and RSV; the remaining 386 samples and all of the 98 healthy controls were identified as negative. Subsequently, 349 infectious patients and 98 healthy participants were applied for 16S rRNA gene sequencing. We randomly selected 10 oropharyngeal samples for metagenomic sequencing from FluA, FluB, and FluA/FluB co-infected patients and healthy participants, respectively (*n* = 40).

### Isolation of DNA and next-generation sequencing

DNA (including negative control samples) was isolated using the Magigene DNA Isolation Kit (Magigene Ltd., Guangzhou, China) and analyzed for DNA integrity on an Agilent Bioanalyzer 2100 (Agilent Technologies, CA, USA). DNA extracted from 447 samples (349 infectious patients and 98 healthy participants), negative control samples (1 sampling negative control, 5 DNA extraction controls, and 3 PCR amplification controls), and together with the mock community as positive controls were further conducted to PCR amplification targeting the V4 region with primers (515F, 5′-GTGCCAGCMGCCGCGGTAA-3′; 806R, 5′-GGACTACHVGGGTWTCTAAT-3′) to generate the sequencing library. Amplifications and purification of PCR products were performed as in the previous study [18], and final libraries were then sequenced on the NovaSeq 6000 platform (Illumina, Inc., CA, USA) with 250bp paired-end reads generated. Overall, the average clean reads of 16S rRNA gene sequencing of specimens in this study were 236,620 (> 90,000 total reads per sample), and the average number of bacterial reads available for downstream analysis was 58,318 (> 20,000 total reads per sample) (Additional file 2: Fig. S2A-B).

A set of 40 samples were subjected to metagenomic sequencing. The libraries were generated using NEB Next® Ultra™ DNA Library Prep Kit for Illumina® (New England Biolabs, MA, USA) with index codes added. The library quality was further assessed on the Qubit 4.0 Fluorometer (Life Technologies, NY, USA) and the Qsep400 High-Throughput Nucleic Acid Protein Analysis System (Houze Biological Technology Co, Hangzhou, China) system. Finally, high-quality libraries were acquired and sequenced on a single sequencing run on the NovaSeq 6000 platform (Illumina, Inc., CA, USA) with 150 bp paired-end reads (> 3 G raw data per sample) generated. The average clean reads of metagenomic sequencing of specimens in this study were 76,576,665 (> 30 million total reads per sample), and the average number of bacterial reads available for downstream analysis was 19,938,678 (> 6 million total reads per sample) (Additional file 2: Fig. S2C).

### Data analysis

#### 16S rRNA gene analysis

Raw “fastq” files were demultiplexed and processed using tools available in QIIME 2 (v2019.7) [32] with a Divisive Amplicon Denoising Algorithm (DADA) 2-based pipeline [33]. Afterward, a feature table containing amplicon sequence variants (ASVs) and associated abundances was generated based on forwarding reads. Representative sequence sets for each dada2 sequence variant were used for taxonomy classification with a Naive Bayes classifier. Sequences were then classified against SILVA (v132) [34]. Finally, alpha- and beta-diversity measurements were performed using an even sampling depth of 20,000 sequences per sample.

#### Negative and positive control assessment

We performed the PCR amplification of the V4 region within 16S rRNA gene for the sampling negative control (*n* = 1), DNA extraction controls (*n* = 5), and PCR amplification controls (*n* = 3). PCR target fragments were not detected in all negative controls and the concentration of purified products was well below the detection limit of the Qubit 4.0 fluorometer. We continued the process with library construction, but the library failed to pass the control samples and were therefore not sequenced. One set of customized mock community was used, which contained purified isolates of two species, a gram-negative bacillus *Escherichia coli* and a gram-positive coccus *Enterococcus faecalis*. Both strains were grown in culture until the OD value was equal to 1.0; 1 ml of each bacterial solution was taken and mixed well as a mock community. Among them, the proportion of *E. coli* in the mock community measured by qPCR was 55.84%, and the proportion of *Enterococcus faecalis* was 44.16%. In our 16S rRNA gene sequencing results, the reads were classified into genera *Escherichia-Shigella* accounting for 56.64%, *Enterococcus* accounting for 40.35%, and only 3.01% of unclassified or other genus sequences outside the mock community.

#### Metagenomics analysis

Sequencing raw data pre-processing was performed by KneadData (v0.7.4, http://huttenhower.sph.harvard.edu/kneaddata), which integrates the tools FastQC (v0.11.9) [35], Trimmomatic (v0.39) [36], and Bowtie2 (v2.3.5.1) [37], to do quality check, quality filtering, and host sequences decontamination, respectively. Taxonomic classification of the resulting reads was performed using MetaPhlAn2 (v2.0) [38]. Metabolic pathway abundance was determined using the HUMAnN2 (v0.9.9) [39] pipeline, and 324 microbial pathways were mapped based on MetaCyc metabolic pathway database. Characterization of antibiotic resistance genes was determined by identifying the annotated markers of the Antibiotic Resistance Database (ARDB) [40] from the metagenomic database using the ShortBRED (v0.9.4) [41] pipeline. The Growth Rate InDex (GRiD) algorithm (v1.3) [42] was used to estimate the growth rates of target bacteria. The microbes in the human respiratory tract, such as opportunistic pathogenic including *Streptococcus pneumoniae* and *Staphylococcus aureus*, and potentially pathogenic bacteria including *Escherichia coli*, *Fusobacterium nucleatum*, *Haemophilus sputorum*, *Mycoplasma pneumoniae*, *Porphyromonas gingivalis*, *Treponema denticola*, and *Treponema medium*, which previously reported [25, 26, 43–47] were summarized as pathogenic species.

#### Microbial diversity

Shannon diversity index was used to characterize alpha-diversity using phyloseq (v1.40.0) [48] package in R (v4.0.5) [49]. Bray-Curtis distance dissimilarities were calculated, and principal coordinates analysis (PCoA) was generated to measure the beta-diversity among samples with phyloseq (v1.40.0).

#### Random Forest analysis

Random Forest regression was used to predict infections based on bacterial abundances using the randomForest package (v4.7-1.1) [50], and the receiver operating characteristic curves (ROC) for each categorical group were plotted. The area under the curve (AUC) was calculated using the pROC (v1.18.0) [51] package in R (v4.0.5). In the random forest model with 100 resamplings, taxa with significant Mean Decrease Accuracy (*P* < 0.05) were considered “important feature,” and the Top-10 taxa with higher value of Mean Decrease Accuracy for each categorical group were applied for statistical analysis.

#### Microbial interaction network

Spearman was used for network inference. The co-presence and mutually exclusive networks were analyzed using the Network analyzer tool of Cytoscape (v3.8.0) [52] and outputted for further analysis, together with node and edge tables for the whole association network.

### Statistical analysis

The distance matrix between microbiota communities was tested by permutational multivariate analysis of variance (PERMANOVA) using vegan (v2.6.2) [53] package in R (v4.0.5). Linear discriminant analysis (LDA) effect size (LEfSe) was conducted on Galaxy (v1.0) [54] for each microbiota paired group to determine significantly different abundances of bacterial taxa, and the parameters were set with the default *P* value (*P* < 0.05) and an LDA score of 2.0. Principal component analysis (PCA) was used to reduce the data dimensionality with ggbiplot (v0.55) [55] package. Spearman correlation analysis was performed using dplyr (v1.1.2) [56] package for network inference among the abundance of bacterial antibiotic resistance genes (ARGs), microbial pathways, and microbes. Differences between categorical groups in the abundance of specific genera were determined by Kruskal-Wallis test as well as Wilcoxon rank-sum test with subsequent Bonferroni correction, with *P* < 0.05 considered significant, respectively, and the *P*-value generated by statistics between healthy and each infectious group was used for Bonferroni correction. Kruskal–Wallis test, Wilcoxon rank-sum test, and Bonferroni were performed with ggpubr (v0.6.0) [57] and R stats (v4.2.2) [49] package, and ggplot2 (v3.4.1) [58] package was used for data visualization.

## Results

### Demographic characteristics of the participants with upper respiratory virus infection

**Fig. 1:**
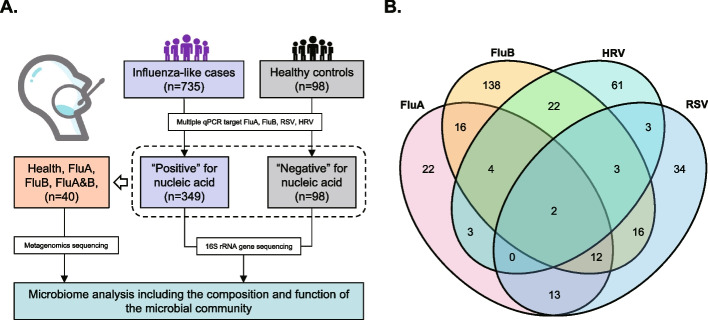
Study flow and virus infection determination. **A** Diagram for the study design. **B** Venn diagram revealed clinical coinfections of different respiratory viruses

From March to August 2019, a total of 735 individuals with influenza-like symptoms were included in this study. The multiplex RT-PCR was used for FluA, FluB, HRV, or RSV verification (Additional file 1: Table S1). As a result, we found that 255 cases were single positive for any of the four viruses, and 94 cases were identified as multiple infections. Also, we recruited 98 healthy participants as controls who were reviewed as negative for viral infection (Fig. 1A, B). The demographics and infection characteristics are summarized in Table 1.

### Viral infections are associated with disorders of specific characteristics of the upper respiratory microbiota

**Fig. 2:**
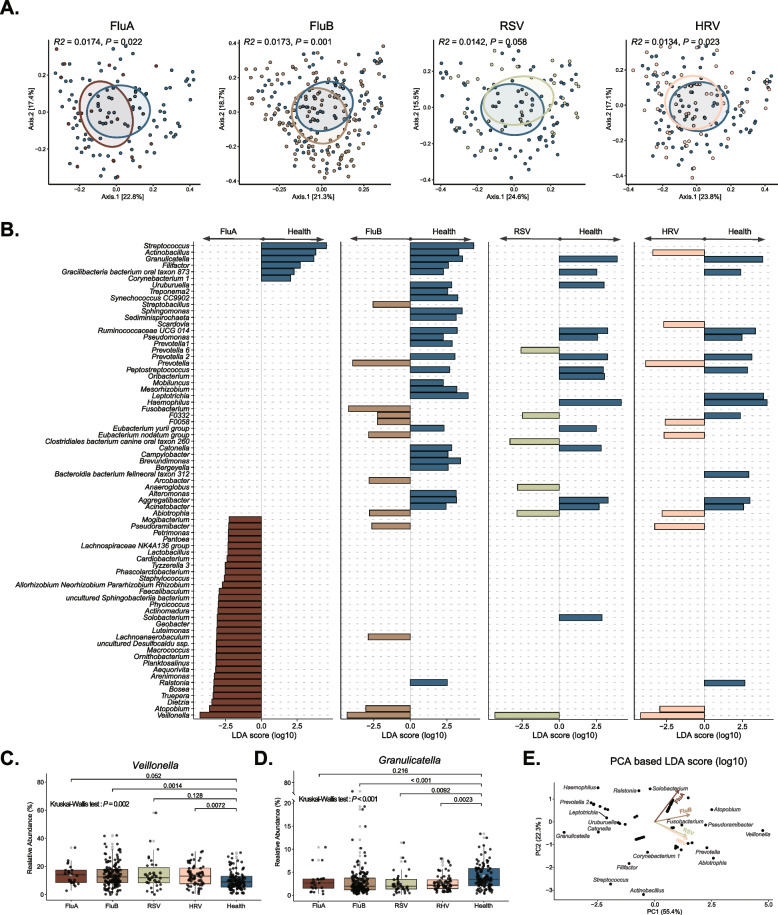
Viral infections correlate to specific upper respiratory tract microbiota disorders. **A** PCoA based on Bray-Curtis distances was analyzed, and PERMANOVA was used to evaluate the variation of microbial composition between the virus-positive and healthy groups. **B** LEfSe was used to calculate the difference between genera between virus-positive and healthy groups and take the union of the genera to show the enrichment features. **C**, **D** Differences in the relative abundance of *Veillonella* and *Granulicatella* between virus-positive and healthy groups (Kruskal-Wallis test as well as Wilcoxon rank-sum test with subsequent Bonferroni correction). **E** PCA was performed based on the LDA values of the different genera, and Biplot was used to evaluate the association between the infectious group (arrows) and genera (points)

To assess the connection of viral infections with the URT microbiota, we performed PCoA and PERMANOVA analysis based on Bray-Curtis distances to reveal the compositional difference between these microbiomes. The result showed that all types of viruses, except RSV, exhibited significant changes in the composition of the oropharyngeal microbiota compared to healthy individuals (Fig. 2A). We further used LEfSe to identify the characteristics of the individual differences in microbiota compositions between the four single virus infections and healthy individuals (Additional file 1: Table S2). We found that *Veillonella* was significantly enriched in the participants with either FluA, FluB, HRV, or RSV infections compared to healthy individuals. In contrast, *Granulicatella* was less abundant in participants with any type of viral infection than in healthy controls (Fig. 2B-D). PCA analysis on LDA values of differential groups showed that the FluA infection exhibits unique features, such as the enrichment of *Solobacterium* and *Ralstonia*, compared to other types of viral infections. Notably, the oropharyngeal microbiota with FluA infection was distinct from that in RSV and HRV groups, which showed similar patterns, whereas the change in microbial compositional characteristics upon FluB infection alone fell between these two (Fig. 2E).

**Fig. 3:**
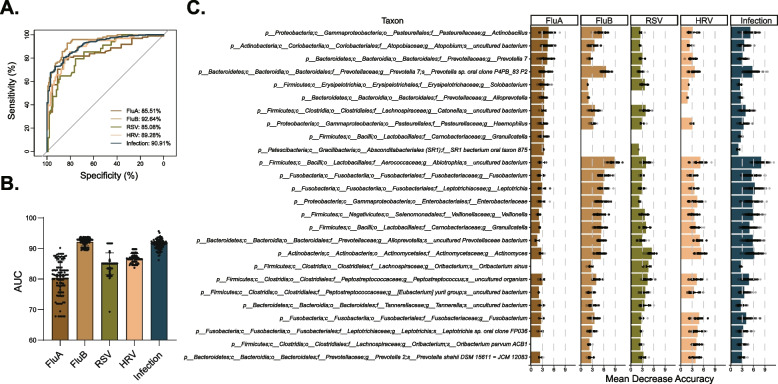
Various viral infections are associated with specific signatures of microbiome disruption. **A** The random forest model was used to predict the participants infected with FluA (*n* = 22), FluB (*n* = 138), RSV (*n* = 34), HRV (*n* = 61), or any virus (*n* = 349) from healthy (*n* = 98), and the ROC was calculated. **B** All samples of the five types were selected respectively; the selected samples and healthy samples were used for random (*n* = 100) under-sampling to fit a random forest model, and the AUC was calculated to evaluate the prediction performance. **C** The Top10-taxa with a higher value of Mean Decrease Accuracy from each categorical group was used for analysis

These findings prompted us to ponder whether the dynamic characteristics of these microbiomes could indicate viral infection or even the type of infection. We then measured the effectiveness of the oropharyngeal microbiome in distinguishing participants with specific viral infections from healthy individuals using a random-forest classification model. We first found that a microbial classifier can effectively identify an infection state. In addition, the area under the receiver operating characteristic curve (AUC) of the FluB (AUC up to 92.64%) group was more extensive than that in the FluA (AUC = 85.51%), RSV (AUC = 85.08%), and HRV (AUC = 89.28%) groups (Fig. 3A, B), indicating that our model most readily distinguishes FluB infections among the four viruses included here. To further characterize the specific species of each microbial classifier, we made a heatmap to show ASVs with significant Mean Decrease Accuracy, which can distinguish infection from non-infection (healthy state) (Fig. 3C). The ASV from genera *Granulicatella*, which serve as a FluA-specific biomarker, was unable to distinguish patients with FluB, RSV, and HRV from participants in this study cohort (Fig. 3C and Additional file 1: Table S3). Our results reveal the value of oropharyngeal bacterial communities for studying a wide range of clinical viral infections; of particular note is that this microbial classifier can effectively distinguish influenza infection, which highlights the association between dysbiosis and specific viral infection.

### Influenza virus infection is linked with increased diversity and functional changes in the upper respiratory microbiota

**Fig. 4:**
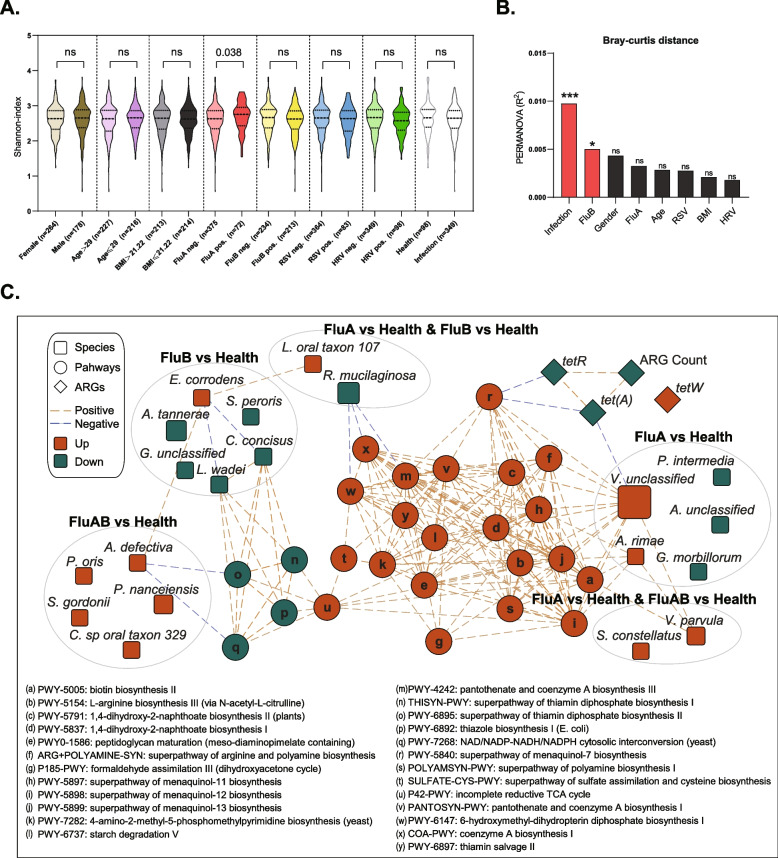
Influenza virus infection is associated with increased diversity and functional changes in the upper respiratory tract microbiota. **A** Differences in alpha-diversity between infected and healthy participants (Wilcoxon rank-sum test). **B** Differences in beta-diversity between infected and healthy participants (PERMANOVA in R with “vegan” package). **C** The Spearman interaction network of differential pathways, differential ARGs, ARGs count, and differential species among the indicated groups were constructed

We analyzed the microbial characteristics of populations with gender, age, BMI, and viral infection types and found that only FluA-positive patients exhibited higher alpha diversity of URT microbiota (Fig. 4A). However, only FluB-positive patients showed a significant difference in beta diversity (Fig. 4B and Additional file 2: Fig. S3). These results above revealed a closer connection between influenza virus infections and URT microbiota, rather than HRV or RSV.

We further investigated the functional and pathogenicity characteristics of the oropharyngeal microbiota associated with influenza virus infection based on the shotgun metagenomics sequencing data. We found that compared to healthy participants, FluA alone infection and FluA/FluB co-infection groups showed enrichment for multiple functional pathways, such as biotin biosynthesis II, L-arginine biosynthesis III (via N-acetyl-L-citrulline) (Additional file 2: Fig. S4A). We also found a significantly lower diversity of drug resistance genes in FluA-positive patients than in healthy participants; nevertheless, either FluA, FluB, or co-infection groups showed higher levels of the tetracycline resistance gene *tetW* (Additional file 2: Fig. S4B-C). Metagenomics sequencing results supported that FluA infection is associated with the significant enrichment of *Veillonella* and *Atopobium*, including species *Veillonella unclassified*, *Veillonella parvula*, and *Atopobium rimae* (Additional file 2: Fig. S4D). We further built an interaction network to demonstrate the association between those microbial species that differ in influenza-infected individuals compared to healthy individuals and ARG expression and functional pathways (Fig. 4C and Additional file 1: Table S4). We found, for example, the potential link of FluA-mediated *V. parvula* enrichment and its positive association with the functional pathways and tetracycline resistance genes, whereas the underlying mechanism needs further investigation.

### Specific respiratory pathogens exhibit higher growth rates in influenza-infected individuals

Opportunistic pathogenic bacteria of the respiratory tract, such as *S. pneumoniae*, *H. influenzae*, and *S. aureus*, are associated with influenza-associated secondary infection [25, 26]. The potentially pathogenic bacteria of the human respiratory tract from previous reports were summarized [43–47], and nine species (in the blue circles) were identified in our oropharyngeal swabs based on metagenomics sequencing analysis (Additional file 2: Fig. S5A). However, the influence of influenza infection on the relative abundance of these pathogens was modest (Additional file 2: Fig. S5B).

**Fig. 5:**
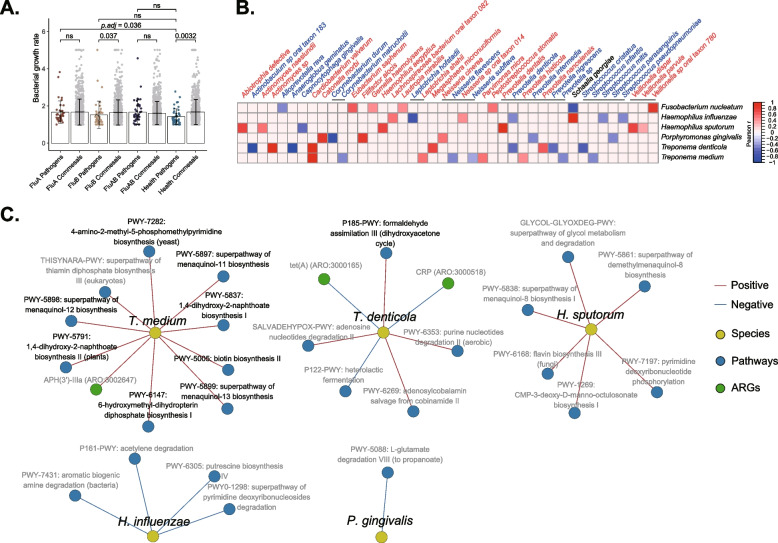
Specific respiratory pathogens exhibit higher growth rates in patients with influenza infection. **A** The growth rate values of all detected potential pathogens and commensals were used to analyze the difference between infected and healthy groups (Wilcoxon rank-sum test with subsequent Bonferroni correction). **B** The correlations of growth rates between pathogens and commensals were analyzed, and the heatmap showed objects with significant associations. **C** Spearman methods revealed the significant correlations between pathogens and bacterial pathways or antibiotics genes, and the networks were displayed with Cytoscape software

To better understand the potential impact of viral infection on oropharyngeal flora, the Growth Rate InDex (GRiD) algorithm was used to estimate the growth rates of pathogens, including *F. nucleatum*, *H. influenzae*, *H. sputorum*, *P. gingivalis*, *T. denticola* and *T. medium* (Additional file 2: Fig. S6 and Additional file 1: Table S5). The results further showed that the growth rate of pathogenic bacteria was significantly lower in healthy controls compared to the commensal bacteria. However, this inhibition was not observed in the infected individuals, which indicates that the influenza virus infection may create an environment that promotes the growth of pathogens (Fig. 5A). Previous studies have suggested commensal bacteria as an alternative approach to the prevention of pathogen infection due to their acting on the host immune system to induce protective responses, or to inhibit the growth of respiratory pathogens by producing antimicrobial products or signals and competing for nutrients and adhesion sites [59, 60]. Our results confirmed that several commensal species such as *Streptococcus infantis*, *Streptococcus mitis*, and *Corynebacterium durum* were significantly negatively correlated with the growth rate of these listed pathogens, suggesting that these commensal microbes may play a crucial role in colonization resistance (Fig. 5B).

Subsequently, we constructed Spearman association networks among the identified six species of pathogens, functional pathways, and drug resistance genes (|*r*| > 0.5, *P* < 0.05). The results showed that the high growth rate of *T. denticola* was significantly correlated to formaldehyde assimilation III (dihydroxyacetone cycle) and other pathways, and the high growth rate of *T. medium* correlates to the enrichment of biotin biosynthesis II, 1,4-dihydroxy-2-naphthoate biosynthesis II, while *H. sputorum* is related to CMP-3-deoxy-D-manno-octulosonate biosynthesis I (Fig. 5C and Additional file 1: Table S6). In conclusion, FluA-positive participants exhibited significantly enhanced growth rates of specific respiratory pathogens, and colonization resistance, multiple bacterial pathways, and drug resistance may be involved in this process. These findings revealed a close association between FluA infection with microbial function and the underlying mechanisms of secondary bacterial infections warrant further investigation.

## Discussion

Morbidity and mortality in severe epidemics are commonly associated with secondary bacterial infections upon viral infections, which has attracted increasing attention to the relationships between dysbiosis and influenza virus, RSV, or other respiratory acute viruses [61–64]. Previous studies of respiratory viruses and human commensal bacteria had focused on a single identified viral infection, ignoring the complexity of an actual infection [65, 66], and it remained unclear whether this dysbiosis is virus-specific. Our study represented a large cohort study and revealed the virus-specific dysbiosis in URT with complex clinical viral infection types. Intriguingly, FluA, FluB, HRV, and RSV infections formed three distinct patterns of ecological dysbiosis, indicating that microbiota dysbiosis has specific virus-dependent features. We also observed significant changes in the composition of respiratory microbiota across four viral infection types. According to the characteristics of this virus-specific microbial disorder, we established the microbial classifiers upon different infection types using a machine learning model and revealed the unique co-occurrence ASVs from *Atopobium* and *Prevotella 7* in FluA and ASVs from *Bergeyella* and *Prevotella aurantiaca* in FluB. These unique features were uncovered to help us for a better understanding of virus pathogenesis and precision therapy. In addition, influenza A was associated with changes in the diversity in the URT microbiome in our study. A previous study reported a significant increase in oropharyngeal microbiota diversity in patients with H7N9 influenza, in particular, H7N9 patients with secondary bacterial pneumonia [12]. This suggests an underlying mechanism for influenza-associated secondary bacterial infections.

A common dysbiosis feature was observed, in which, *Veillonella* was significantly enriched in all types of viral infection groups compared to healthy participants. *Veillonella* was previously reported as the causative agent of chronic anaerobic pneumonia [67]. Furthermore, *Veillonella* has been shown to be over-represented in the URT, bronchoalveolar lavage fluid, or lung tissues of COVID-19 patients [68–70], and the occurrence of *Veillonella* is closely associated with the enrichment of multiple pathogens such as *S. aureus* and *Prevotella melaninogenica* [71, 72]. These results suggest that *Veillonella* may serve as an indicator of dysbiosis following viral infections in the URT and that we should focus on its role and potential mechanisms in terms of its pro-inflammatory capacity and bacterial interactions.

Previous studies have reported significant differences in nasopharyngeal microbial profiles between HRV and RSV infections [73], and the oropharyngeal microbial differences among FluA, FluB, HRV, and RSV infections were observed in this study, which indicate universal changes in the microbiome of the URT during acute respiratory virus infections. Recent studies on the relationship between the upper respiratory microbiome and respiratory infections have mainly relied on 16S rRNA sequencing rather than metagenomic studies [62, 66, 74], which are limited by the lack of resolution on the strain or subspecies levels as functional genes (such as drug resistance genes) and pathways. In our study, we combined metagenomic sequencing with 16S rRNA analysis to improve the resolution of the analysis. Our metagenomic data further suggested that enriched *V. parvula* abundance in FluA and FluA/FluB co-infected patients is associated with higher biotin synthesis capacity. The biotin synthetic pathway is considered one of the unique biological pathways in bacteria and has been proposed as a promising antimicrobial target [75]. Our previous study showed that *Veillonella* is able to migrate from the oral cavity to the lungs and is associated with inflammation and impaired function in the LRT [76]. Together with this study, we can rationally infer that local airway microbiota dysbiosis may be impacted by the viral infection that initially occurs in the oral cavity and subsequently impacts distant microbiota through the oral-lung axis. Therefore, manipulation of the oropharyngeal microbiome may be a selective prevention strategy.

In addition to identifying the characteristics of respiratory dysbiosis associated with different viral infections, we further proposed that the increased growth rate of pathogenic bacteria is the crucial process of influenza viral infections, which may directly mediate secondary bacterial infections in the lower respiratory tract. Bioinformatic tools such as MetaPhlAn [38] and Kraken [77] are able to accurately quantify pathogenic bacteria abundance based on metagenomic sequencing data; however, the growth rates of species, particularly pathogens, are of greater concern, especially in the early stages of secondary infection, which may directly contribute to disease progression [42, 78]. More importantly, we can better understand microbial interactions by estimating the growth rate differences between commensals and pathogens. Our study aimed to reveal the growth patterns of commensal and pathogens upon different respiratory virus infections. We found that the growth rates of pathogenic bacteria were significantly increased only in patients infected with FluA or FluB. In addition, some commensals, such as *S. mitis*, may activate the colonization resistance to pathogenic bacteria during viral infection. Several previous studies have found that *Streptococcus* spp*.* abundance in URT was correlated with lower susceptibility to influenza A (H3N2) and influenza B infection [79]. Among healthy young adults vaccinated with live attenuated influenza vaccine, *S. infantis* was positively associated with influenza H1 immunoglobulin A (IgA) titers [80]. These results suggest that some members of *Streptococcus* may have probiotic potential against respiratory virus infections.

## Conclusions

We found that respiratory viral infections correlate to viral type-specific disruption of the upper respiratory microbiota, and *Veillonella* was identified as a prominent biomarker for any type of the four respiratory virus infections. Intriguingly, influenza infections are uniquely associated with enhanced microbial diversity and growth rates of specific pathogens in URT. We proposed that pre-changes in the oropharyngeal microbiome are potentially indicative of respiratory viruses-induced secondary bacterial infections, which provides a better understanding of complications of upper respiratory tract infection. While further investigations are needed to translate these results into potential clinical and public health applications, our findings demonstrated a critical role of microbiome perturbations in bridging the link between upper respiratory viral infections and severe secondary infections.

## Supplementary Information

**Additional file 1: Table S1.** List of primers used for multiplex qPCR. **Table S2.** The differential genera between virus-positive and negative groups. **Table S3.** In the random forest model with 100 resamplings, the Mean Decrease Accuracy from group 1 and group 2 was used for the Wilcoxon test. **Table S4.** The Spearman interaction of differential pathways, differential ARGs, ARGs count, and differential species in the FluA group and healthy controls. **Table S5.** The growth rate of potential pathogens in situ calculated by GRID algorithm. **Table S6.** The Spearman interaction of all pathways, ARGs, and pathogens.

**Additional file 2: Figure S1.** Multiple qPCR standard curves for FluA, FluB, RSV, and HR. **Figure S2.** Reads count and proportions of microbiota in sequencing profiles. (A) Microbial reads count in 16S rRNA gene sequencing data. (B) The proportions of microbiota in 16S rRNA gene sequencing data. (C) Microbial and human reads count in metagenomic sequencing data. **Figure S3.** The variation of microbial composition between virus-positive and negative groups. PCoA based on Bray–Curtis distances was analyzed, and PERMANOVA was used to evaluate the variation. **Figure S4.** The differential genera, pathway, and ARGs between health and FluA, FluB or FluAB group. (A) Compared to healthy controls, the functional pathways (MetaCyc database) with significant differences in virus infection were calculated. The number and color represent the fold change relative to the control group. Blank blocks indicate no significant difference. (B) The number of antibiotic resistance genes (ARGs) based on metagenomic data was used to evaluate the diversity of ARGs, and the variation between the infected and healthy groups was counted by Kruskal–Wallis test and Wilcoxon test with subsequent Bonferroni correction. (C) Significantly different resistance genes compared to healthy controls were shown. (D) LEfSe was used to calculate the different genera between healthand FluA, FluB, orFluAB participants, respectively. **Figure S5.** The relative abundance difference of potential pathogens between infected and healthy subjects. (A) Nine pathogenic species (in the blue circle) were identified in the oropharyngeal samples based on metagenomics sequencing analysis. (B) Kruskal-Wallis test and Wilcoxon test was used to calculate the relative abundance difference of nine pathogens between the infected and the healthy subjects. **Figure S6.** The growth rate of potential pathogens (in the blue circle) was detected based on metagenomics sequencing analysis.

## Data Availability

The datasets supporting the conclusions of this article are available in the PRJNA801796 repository, unique persistent identifier and hyperlink to datasets at http://www.ncbi.nlm.nih.gov/sra. The R scripts used to generate the results in this manuscript are available in the following Git repository: https://github.com/TaoDing/URTI_microbiota.
