# Supplementary material for: Unique microbial landscape in the human oropharynx during different types of acute respiratory tract infections
Source: Microbiome. 2023 Jul 24;11:157. doi: 10.1186/s40168-023-01597-9 (PMC10364384; doi:10.1186/s40168-023-01597-9)
Supplement: Supplementary file 2 — Additional file 1: Table S1. List of primers used for multiplex qPCR. Table S2. The differential genera between virus-positive and negative groups. Table S3. In the random forest model with 100 resamplings, the Mean Decrease Accuracy from group 1 and group 2 was used for the Wilcoxon test. Table S4. The Spearman interaction of differential pathways, differential ARGs, ARGs count, and differential species in the FluA group and healthy controls. Table S5. The growth rate of potential pathogens in situ calculated by GRID algorithm. Table S6. The Spearman interaction of all pathways, ARGs, and pathogens. [file 40168_2023_1597_MOESM1_ESM.pdf]

Table S1. List of primers used for multiplex qPCR.

Target virus, Gene	Primer and probe sequences	Fluorescent and quenched signal
Influenza A virus, M1	F: GACCRATCCTGTCACCTCTGAC<br>R: AGGGCATTYTGGACAAAKCGTCTA<br>P: TGCAGTCCTCGCTCACTGGGCACG	5’-FAM, 3’-BHQ1
Influenza B virus, HA	F: AAATACGGTGGATTAAATAAAAGCAA<br>R: CCAGCAATAGCTCCGAAGAAA<br>P: CACCCATATTGGGCAATTTCCTAT	5’-HEX, 3’-BHQ1
RSV, matrix protein	F: GCAAATATGGAAACATACGTGAACA<br>R: GCACCCATATTGTWAGTGATGCA<br>P: CTTCACGAAGGCTCCACATACACAGCWG	5’-TexasRed, 3’-BHQ2
Rhinovirus, 5’ UTR	F1: GGTGTGAAGAGCCSCRTGTGCT<br>F2: GGTGTGAAGACTCGCATGTGCT<br>F3: GGGTGYGAAGAGYCTANTGTGCT<br>R3: GGACACCCAAAGTAGTYGGTYC<br>P: CCGGCCCCTGAATGYGGCTAAYC	5’-CY5, 3’-MGB

Table S2. The differential genera between virus-positive and negative groups.

**Difference genera in LEfSe analysis**	**Enriched groups**	***LDA* value**	***P* value**	**Comparison between groups**
Aequorivita	FluA	3.192	0.035	FluA vs Health
Truepera	FluA	3.372	0.035	FluA vs Health
Staphylococcus	FluA	2.600	0.029	FluA vs Health
uncultured Desulfocaldu ssp.	FluA	3.148	0.035	FluA vs Health
Cardiobacterium	FluA	2.399	0.046	FluA vs Health
Planktosalinus	FluA	3.164	0.035	FluA vs Health
Tyzzerella 3	FluA	2.518	0.003	FluA vs Health
Faecalibaculum	FluA	2.948	0.035	FluA vs Health
Allorhizobium-Neorhizobium-Pararhizobium-Rhizobium	FluA	2.715	0.035	FluA vs Health
Ralstonia	FluA	3.324	0.024	FluA vs Health
Arenimonas	FluA	3.299	0.035	FluA vs Health
Actinomadura	FluA	3.046	0.035	FluA vs Health
Lactobacillus	FluA	2.333	0.002	FluA vs Health
Petrimonas	FluA	2.296	0.031	FluA vs Health
Geobacter	FluA	3.085	0.035	FluA vs Health
Veillonella	FluA	4.287	0.013	FluA vs Health
Dietzia	FluA	3.460	0.035	FluA vs Health
Bosea	FluA	3.330	0.035	FluA vs Health
Pseudoramibacter	FluA	2.291	0.003	FluA vs Health
Lachnospiraceae NK4A136	FluA	2.330	0.029	FluA vs Health
Lachnoanaero baculum	FluA	3.130	0.014	FluA vs Health
Solobacterium	FluA	3.083	0.018	FluA vs Health
Luteimonas	FluA	3.116	0.035	FluA vs Health
Ornithobacterium	FluA	3.161	0.035	FluA vs Health
Uncultured Sphingobacteriia	FluA	2.982	0.035	FluA vs Health
Mogibacterium	FluA	2.244	0.003	FluA vs Health
Phycicoccus	FluA	3.016	0.035	FluA vs Health
Atopobium	FluA	3.623	<0.001	FluA vs Health
Phascolarcto bacterium	FluA	2.530	0.027	FluA vs Health
Pantoea	FluA	2.314	0.029	FluA vs Health
Macrococcus	FluA	3.150	0.035	FluA vs Health
Gracilibacteria bacterium oral taxon 873	Health	2.282	0.022	FluA vs Health
Filifactor	Health	2.722	0.048	FluA vs Health
Actinobacillus	Health	3.801	<0.001	FluA vs Health
Streptococcus	Health	4.551	0.043	FluA vs Health
Corynebacterium 1	Health	2.048	0.043	FluA vs Health
Granulicatella	Health	3.669	0.050	FluA vs Health
Abiotrophia	FluB	2.791	<0.001	FluB vs Health

Streptobacillus	FluB	2.544	0.045	FluB vs Health
Veillonella	FluB	4.312	<0.001	FluB vs Health
Fusobacterium	FluB	4.218	0.014	FluB vs Health
F0058	FluB	2.250	0.030	FluB vs Health
Pseudoramibacter	FluB	2.627	0.004	FluB vs Health
Eubacterium nodatum group	FluB	2.836	0.022	FluB vs Health
Prevotella	FluB	3.929	0.014	FluB vs Health
Lachnoanaerobaculum	FluB	2.874	0.013	FluB vs Health
F0332	FluB	2.237	0.037	FluB vs Health
Atopobium	FluB	3.038	0.001	FluB vs Health
Arcobacter	FluB	2.812	0.037	FluB vs Health
Leptotrichia	Health	3.964	<0.001	FluB vs Health
Sphingomonas	Health	3.544	0.017	FluB vs Health
Brevundimonas	Health	3.439	0.039	FluB vs Health
Campylobacter	Health	2.574	0.005	FluB vs Health
Gracilibacteria bacterium oral taxon 873	Health	2.258	0.004	FluB vs Health
Ruminococcaceae UCG_014	Health	3.203	0.010	FluB vs Health
Prevotella 1	Health	2.861	0.033	FluB vs Health
Prevotella 2	Health	3.050	0.025	FluB vs Health
Alteromonas	Health	3.139	0.017	FluB vs Health
Ralstonia	Health	2.530	0.001	FluB vs Health
Sediminispirochaeta	Health	3.145	0.039	FluB vs Health
Mobiluncus	Health	2.252	0.017	FluB vs Health
Mesorhizobium	Health	3.180	0.017	FluB vs Health
Catonella	Health	2.828	<0.001	FluB vs Health
Peptostreptococcus	Health	2.681	0.002	FluB vs Health
Aggregatibacter	Health	3.166	0.008	FluB vs Health
Filifactor	Health	2.610	0.008	FluB vs Health
Bergeyella	Health	2.589	0.022	FluB vs Health
Synechococcus CC9902	Health	3.251	0.039	FluB vs Health
Treponema 2	Health	2.556	0.010	FluB vs Health
Actinobacillus	Health	3.313	<0.001	FluB vs Health
Pseudomonas	Health	2.250	<0.001	FluB vs Health
Acinetobacter	Health	2.447	0.040	FluB vs Health
Streptococcus	Health	4.334	0.014	FluB vs Health
Uruburuella	Health	2.831	<0.001	FluB vs Health
Granulicatella	Health	3.582	<0.001	FluB vs Health
Eubacterium yurii group	Health	2.308	0.009	FluB vs Health
Leptotrichia	Health	3.901	0.013	HRV vs Health
Gracilibacteria bacterium oral taxon 873	Health	2.382	0.005	HRV vs Health
Bacteroidia bacterium feline oral taxon 312	Health	2.917	0.050	HRV vs Health

Ruminococcaceae UCG_014	Health	3.351	<0.001	HRV vs Health
Prevotella 2	Health	3.121	0.001	HRV vs Health
Ralstonia	Health	2.666	0.012	HRV vs Health
Peptostreptococcus	Health	2.829	<0.001	HRV vs Health
Aggregatibacter	Health	2.993	0.010	HRV vs Health
Pseudomonas	Health	2.472	0.001	HRV vs Health
F0332	Health	2.362	0.005	HRV vs Health
Acinetobacter	Health	2.579	0.011	HRV vs Health
Haemophilus	Health	4.126	0.025	HRV vs Health
Granulicatella	Health	3.832	0.001	HRV vs Health
Abiotrophia	HRV	2.785	<0.001	HRV vs Health
Veillonella	HRV	4.206	0.002	HRV vs Health
F0058	HRV	2.589	0.039	HRV vs Health
Actinobacillus	HRV	3.400	0.036	HRV vs Health
Pseudoramibacter	HRV	3.281	0.001	HRV vs Health
Scardovia	HRV	2.681	0.013	HRV vs Health
Eubacterium nodatum group	HRV	2.662	0.033	HRV vs Health
Prevotella	HRV	3.876	0.021	HRV vs Health
Atopobium	HRV	2.935	0.018	HRV vs Health
Gracilibacteria bacterium oral taxon 873	Health	2.522	0.015	RSV vs Health
Ruminococcaceae UCG_014	Health	3.268	0.031	RSV vs Health
Prevotella 2	Health	3.243	0.005	RSV vs Health
Oribacterium	Health	3.041	0.009	RSV vs Health
Catonella	Health	2.823	0.003	RSV vs Health
Peptostreptococcus	Health	2.960	<0.001	RSV vs Health
Aggregatibacter	Health	3.295	0.003	RSV vs Health
Pseudomonas	Health	2.580	0.001	RSV vs Health
Solobacterium	Health	2.875	0.003	RSV vs Health
Acinetobacter	Health	2.678	0.039	RSV vs Health
Uruburuella	Health	3.015	0.033	RSV vs Health
Haemophilus	Health	4.178	0.010	RSV vs Health
Granulicatella	Health	3.897	0.002	RSV vs Health
Eubacterium yurii group	Health	2.495	0.009	RSV vs Health
Prevotella 6	RSV	2.574	0.038	RSV vs Health
Clostridiales bacterium canine oral taxon 260	RSV	3.318	0.004	RSV vs Health
Abiotrophia	RSV	2.843	<0.001	RSV vs Health
Anaeroglobus	RSV	2.805	<0.001	RSV vs Health
Veillonella	RSV	4.338	0.034	RSV vs Health
F0332	RSV	2.469	0.001	RSV vs Health

Table S3, In the random forest model with 100 resamplings, the Mean Decrease Accuracy from group 1 and group 2 was used for the Wilcoxon test.

Taxon	group1	group2	*p*	*p.adj*	*p.signif*	method
p__Firmicutes;c__Bacilli;o__Lactobacillales;f__Carnobacteriaceae;g__Granulicatella	FluA	FluB	3.14E-17	2.80E-16	****	Wilcoxon
p__Firmicutes;c__Bacilli;o__Lactobacillales;f__Carnobacteriaceae;g__Granulicatella	FluA	RSV	0.002479	0.0074	**	Wilcoxon
p__Firmicutes;c__Bacilli;o__Lactobacillales;f__Carnobacteriaceae;g__Granulicatella	FluA	HRV	0.013809	0.028	*	Wilcoxon
p__Firmicutes;c__Bacilli;o__Lactobacillales;f__Carnobacteriaceae;g__Granulicatella	FluA	Infection	4.25E-09	3.00E-08	****	Wilcoxon
p__Firmicutes;c__Bacilli;o__Lactobacillales;f__Carnobacteriaceae;g__Granulicatella	FluB	RSV	2.01E-11	1.60E-10	****	Wilcoxon
p__Firmicutes;c__Bacilli;o__Lactobacillales;f__Carnobacteriaceae;g__Granulicatella	FluB	HRV	2.15E-18	2.20E-17	****	Wilcoxon
p__Firmicutes;c__Bacilli;o__Lactobacillales;f__Carnobacteriaceae;g__Granulicatella	FluB	Infection	0.000187	0.00093	***	Wilcoxon
p__Firmicutes;c__Bacilli;o__Lactobacillales;f__Carnobacteriaceae;g__Granulicatella	RSV	HRV	0.138501	0.14	ns	Wilcoxon
p__Firmicutes;c__Bacilli;o__Lactobacillales;f__Carnobacteriaceae;g__Granulicatella	RSV	Infection	0.001067	0.0043	**	Wilcoxon
p__Firmicutes;c__Bacilli;o__Lactobacillales;f__Carnobacteriaceae;g__Granulicatella	HRV	Infection	3.64E-07	2.20E-06	****	Wilcoxon
p__Bacteroidetes;c__Bacteroidia;o__Bacteroidales;f__Prevotellaceae;g__Prevotella 7;s__Prevotella sp. oral clone P4PB_83 P2	FluA	FluB	1.11E-26	1.00E-25	****	Wilcoxon
p__Bacteroidetes;c__Bacteroidia;o__Bacteroidales;f__Prevotellaceae;g__Prevotella 7;s__Prevotella sp. oral clone P4PB_83 P2	FluA	RSV	8.13E-09	2.40E-08	****	Wilcoxon
p__Bacteroidetes;c__Bacteroidia;o__Bacteroidales;f__Prevotellaceae;g__Prevotella 7;s__Prevotella sp. oral clone P4PB_83 P2	FluA	HRV	0.261253	0.26	ns	Wilcoxon
p__Bacteroidetes;c__Bacteroidia;o__Bacteroidales;f__Prevotellaceae;g__Prevotella 7;s__Prevotella sp. oral clone P4PB_83 P2	FluA	Infection	2.63E-18	1.80E-17	****	Wilcoxon
p__Bacteroidetes;c__Bacteroidia;o__Bacteroidales;f__Prevotellaceae;g__Prevotella 7;s__Prevotella sp. oral clone P4PB_83 P2	FluB	RSV	1.18E-14	7.10E-14	****	Wilcoxon
p__Bacteroidetes;c__Bacteroidia;o__Bacteroidales;f__Prevotellaceae;g__Prevotella 7;s__Prevotella sp. oral clone P4PB_83 P2	FluB	HRV	1.70E-31	1.70E-30	****	Wilcoxon
p__Bacteroidetes;c__Bacteroidia;o__Bacteroidales;f__Prevotellaceae;g__Prevotella 7;s__Prevotella sp. oral clone P4PB_83 P2	FluB	Infection	3.87E-11	1.50E-10	****	Wilcoxon
p__Bacteroidetes;c__Bacteroidia;o__Bacteroidales;f__Prevotellaceae;g__Prevotella 7;s__Prevotella sp. oral clone P4PB_83 P2	RSV	HRV	1.78E-08	3.60E-08	****	Wilcoxon
p__Bacteroidetes;c__Bacteroidia;o__Bacteroidales;f__Prevotellaceae;g__Prevotella 7;s__Prevotella sp. oral clone P4PB_83 P2	RSV	Infection	3.47E-14	1.70E-13	****	Wilcoxon
p__Bacteroidetes;c__Bacteroidia;o__Bacteroidales;f__Prevotellaceae;g__Prevotella 7;s__Prevotella sp. oral clone P4PB_83 P2	HRV	Infection	2.73E-23	2.20E-22	****	Wilcoxon
p__Proteobacteria;c__Gammaproteobacteria;o__Pasteurellales;f__Pasteurellaceae;g__Haemophilus	FluA	FluB	0.066916	0.33	ns	Wilcoxon
p__Proteobacteria;c__Gammaproteobacteria;o__Pasteurellales;f__Pasteurellaceae;g__Haemophilus	FluA	RSV	0.002375	0.024	**	Wilcoxon
p__Proteobacteria;c__Gammaproteobacteria;o__Pasteurellales;f__Pasteurellaceae;g__Haemophilus	FluA	HRV	0.008529	0.06	**	Wilcoxon
p__Proteobacteria;c__Gammaproteobacteria;o__Pasteurellales;f__Pasteurellaceae;g__Haemophilus	FluA	Infection	0.203285	0.81	ns	Wilcoxon
p__Proteobacteria;c__Gammaproteobacteria;o__Pasteurellales;f__Pasteurellaceae;g__Haemophilus	FluB	RSV	0.003885	0.031	**	Wilcoxon
p__Proteobacteria;c__Gammaproteobacteria;o__Pasteurellales;f__Pasteurellaceae;g__Haemophilus	FluB	HRV	0.002361	0.024	**	Wilcoxon
p__Proteobacteria;c__Gammaproteobacteria;o__Pasteurellales;f__Pasteurellaceae;g__Haemophilus	FluB	Infection	0.312946	0.94	ns	Wilcoxon
p__Proteobacteria;c__Gammaproteobacteria;o__Pasteurellales;f__Pasteurellaceae;g__Haemophilus	RSV	HRV	0.626976	1	ns	Wilcoxon
p__Proteobacteria;c__Gammaproteobacteria;o__Pasteurellales;f__Pasteurellaceae;g__Haemophilus	RSV	Infection	0.021042	0.13	*	Wilcoxon
p__Proteobacteria;c__Gammaproteobacteria;o__Pasteurellales;f__Pasteurellaceae;g__Haemophilus	HRV	Infection	0.792703	1	ns	Wilcoxon
p__Proteobacteria;c__Gammaproteobacteria;o__Pasteurellales;f__Pasteurellaceae;g__Actinobacillus	FluA	FluB	5.23E-08	2.60E-07	****	Wilcoxon
p__Proteobacteria;c__Gammaproteobacteria;o__Pasteurellales;f__Pasteurellaceae;g__Actinobacillus	FluA	RSV	3.17E-07	1.30E-06	****	Wilcoxon
p__Proteobacteria;c__Gammaproteobacteria;o__Pasteurellales;f__Pasteurellaceae;g__Actinobacillus	FluA	HRV	7.28E-12	5.80E-11	****	Wilcoxon

p__Proteobacteria;c__Gammaproteobacteria;o__Pasteurellales;f__Pasteurellaceae;g__Actinobacillus	FluA	Infection	0.014253	0.029	*	Wilcoxon
p__Proteobacteria;c__Gammaproteobacteria;o__Pasteurellales;f__Pasteurellaceae;g__Actinobacillus	FluB	RSV	1.74E-09	1.20E-08	****	Wilcoxon
p__Proteobacteria;c__Gammaproteobacteria;o__Pasteurellales;f__Pasteurellaceae;g__Actinobacillus	FluB	HRV	6.18E-23	6.20E-22	****	Wilcoxon
p__Proteobacteria;c__Gammaproteobacteria;o__Pasteurellales;f__Pasteurellaceae;g__Actinobacillus	FluB	Infection	0.002755	0.0083	**	Wilcoxon
p__Proteobacteria;c__Gammaproteobacteria;o__Pasteurellales;f__Pasteurellaceae;g__Actinobacillus	RSV	HRV	0.159414	0.16	ns	Wilcoxon
p__Proteobacteria;c__Gammaproteobacteria;o__Pasteurellales;f__Pasteurellaceae;g__Actinobacillus	RSV	Infection	2.46E-08	1.50E-07	****	Wilcoxon
p__Proteobacteria;c__Gammaproteobacteria;o__Pasteurellales;f__Pasteurellaceae;g__Actinobacillus	HRV	Infection	1.53E-16	1.40E-15	****	Wilcoxon
p__Actinobacteria;c__Coriobacteriia;o__Coriobacteriales;f__Atopobiaceae;g__Atopobium;s__uncultured bacterium	FluA	FluB	4.40E-06	3.50E-05	****	Wilcoxon
p__Actinobacteria;c__Coriobacteriia;o__Coriobacteriales;f__Atopobiaceae;g__Atopobium;s__uncultured bacterium	FluA	RSV	0.016368	0.082	*	Wilcoxon
p__Actinobacteria;c__Coriobacteriia;o__Coriobacteriales;f__Atopobiaceae;g__Atopobium;s__uncultured bacterium	FluA	HRV	2.97E-07	2.70E-06	****	Wilcoxon
p__Actinobacteria;c__Coriobacteriia;o__Coriobacteriales;f__Atopobiaceae;g__Atopobium;s__uncultured bacterium	FluA	Infection	4.77E-08	4.80E-07	****	Wilcoxon
p__Actinobacteria;c__Coriobacteriia;o__Coriobacteriales;f__Atopobiaceae;g__Atopobium;s__uncultured bacterium	FluB	RSV	0.097467	0.29	ns	Wilcoxon
p__Actinobacteria;c__Coriobacteriia;o__Coriobacteriales;f__Atopobiaceae;g__Atopobium;s__uncultured bacterium	FluB	HRV	0.000125	0.00075	***	Wilcoxon
p__Actinobacteria;c__Coriobacteriia;o__Coriobacteriales;f__Atopobiaceae;g__Atopobium;s__uncultured bacterium	FluB	Infection	0.435704	0.87	ns	Wilcoxon
p__Actinobacteria;c__Coriobacteriia;o__Coriobacteriales;f__Atopobiaceae;g__Atopobium;s__uncultured bacterium	RSV	HRV	0.481102	0.87	ns	Wilcoxon
p__Actinobacteria;c__Coriobacteriia;o__Coriobacteriales;f__Atopobiaceae;g__Atopobium;s__uncultured bacterium	RSV	Infection	0.073494	0.29	ns	Wilcoxon
p__Actinobacteria;c__Coriobacteriia;o__Coriobacteriales;f__Atopobiaceae;g__Atopobium;s__uncultured bacterium	HRV	Infection	3.13E-05	0.00022	****	Wilcoxon
p__Bacteroidetes;c__Bacteroidia;o__Bacteroidales;f__Prevotellaceae;g__Prevotella 7	FluA	FluB	7.78E-07	5.40E-06	****	Wilcoxon
p__Bacteroidetes;c__Bacteroidia;o__Bacteroidales;f__Prevotellaceae;g__Prevotella 7	FluA	RSV	3.05E-09	2.40E-08	****	Wilcoxon
p__Bacteroidetes;c__Bacteroidia;o__Bacteroidales;f__Prevotellaceae;g__Prevotella 7	FluA	HRV	1.63E-11	1.60E-10	****	Wilcoxon
p__Bacteroidetes;c__Bacteroidia;o__Bacteroidales;f__Prevotellaceae;g__Prevotella 7	FluA	Infection	1.42E-10	1.30E-09	****	Wilcoxon
p__Bacteroidetes;c__Bacteroidia;o__Bacteroidales;f__Prevotellaceae;g__Prevotella 7	FluB	RSV	0.383294	1	ns	Wilcoxon
p__Bacteroidetes;c__Bacteroidia;o__Bacteroidales;f__Prevotellaceae;g__Prevotella 7	FluB	HRV	0.004697	0.028	**	Wilcoxon
p__Bacteroidetes;c__Bacteroidia;o__Bacteroidales;f__Prevotellaceae;g__Prevotella 7	FluB	Infection	0.083102	0.42	ns	Wilcoxon
p__Bacteroidetes;c__Bacteroidia;o__Bacteroidales;f__Prevotellaceae;g__Prevotella 7	RSV	HRV	0.300664	1	ns	Wilcoxon
p__Bacteroidetes;c__Bacteroidia;o__Bacteroidales;f__Prevotellaceae;g__Prevotella 7	RSV	Infection	0.909065	1	ns	Wilcoxon
p__Bacteroidetes;c__Bacteroidia;o__Bacteroidales;f__Prevotellaceae;g__Prevotella 7	HRV	Infection	0.532642	1	ns	Wilcoxon
p__Fusobacteria;c__Fusobacteriia;o__Fusobacteriales;f__Leptotrichiaceae;g__Leptotrichia	FluA	FluB	2.06E-13	1.20E-12	****	Wilcoxon
p__Fusobacteria;c__Fusobacteriia;o__Fusobacteriales;f__Leptotrichiaceae;g__Leptotrichia	FluA	RSV	0.946434	0.95	ns	Wilcoxon
p__Fusobacteria;c__Fusobacteriia;o__Fusobacteriales;f__Leptotrichiaceae;g__Leptotrichia	FluA	HRV	0.014827	0.03	*	Wilcoxon
p__Fusobacteria;c__Fusobacteriia;o__Fusobacteriales;f__Leptotrichiaceae;g__Leptotrichia	FluA	Infection	2.51E-13	1.30E-12	****	Wilcoxon
p__Fusobacteria;c__Fusobacteriia;o__Fusobacteriales;f__Leptotrichiaceae;g__Leptotrichia	FluB	RSV	8.02E-29	8.00E-28	****	Wilcoxon
p__Fusobacteria;c__Fusobacteriia;o__Fusobacteriales;f__Leptotrichiaceae;g__Leptotrichia	FluB	HRV	2.85E-21	2.30E-20	****	Wilcoxon
p__Fusobacteria;c__Fusobacteriia;o__Fusobacteriales;f__Leptotrichiaceae;g__Leptotrichia	FluB	Infection	0.008898	0.027	**	Wilcoxon
p__Fusobacteria;c__Fusobacteriia;o__Fusobacteriales;f__Leptotrichiaceae;g__Leptotrichia	RSV	HRV	6.19E-06	2.50E-05	****	Wilcoxon
p__Fusobacteria;c__Fusobacteriia;o__Fusobacteriales;f__Leptotrichiaceae;g__Leptotrichia	RSV	Infection	3.92E-28	3.50E-27	****	Wilcoxon
p__Fusobacteria;c__Fusobacteriia;o__Fusobacteriales;f__Leptotrichiaceae;g__Leptotrichia	HRV	Infection	7.68E-19	5.40E-18	****	Wilcoxon

p__Firmicutes;c__Clostridia;o__Clostridiales;f__Lachnospiraceae;g__Catonella;s__uncultured bacterium	FluA	FluB	0.546816	1	ns	Wilcoxon
p__Firmicutes;c__Clostridia;o__Clostridiales;f__Lachnospiraceae;g__Catonella;s__uncultured bacterium	FluA	RSV	0.097101	0.58	ns	Wilcoxon
p__Firmicutes;c__Clostridia;o__Clostridiales;f__Lachnospiraceae;g__Catonella;s__uncultured bacterium	FluA	Infection	0.57982	1	ns	Wilcoxon
p__Firmicutes;c__Clostridia;o__Clostridiales;f__Lachnospiraceae;g__Catonella;s__uncultured bacterium	FluB	RSV	0.198837	0.95	ns	Wilcoxon
p__Firmicutes;c__Clostridia;o__Clostridiales;f__Lachnospiraceae;g__Catonella;s__uncultured bacterium	FluB	Infection	0.883474	1	ns	Wilcoxon
p__Firmicutes;c__Clostridia;o__Clostridiales;f__Lachnospiraceae;g__Catonella;s__uncultured bacterium	RSV	Infection	0.190396	0.95	ns	Wilcoxon
p__Firmicutes;c__Negativicutes;o__Selenomonadales;f__Veillonellaceae;g__Veillonella	FluA	FluB	1.63E-11	9.80E-11	****	Wilcoxon
p__Firmicutes;c__Negativicutes;o__Selenomonadales;f__Veillonellaceae;g__Veillonella	FluA	RSV	1.72E-09	6.90E-09	****	Wilcoxon
p__Firmicutes;c__Negativicutes;o__Selenomonadales;f__Veillonellaceae;g__Veillonella	FluA	HRV	1.24E-08	3.70E-08	****	Wilcoxon
p__Firmicutes;c__Negativicutes;o__Selenomonadales;f__Veillonellaceae;g__Veillonella	FluA	Infection	1.66E-11	9.80E-11	****	Wilcoxon
p__Firmicutes;c__Negativicutes;o__Selenomonadales;f__Veillonellaceae;g__Veillonella	FluB	RSV	5.24E-19	3.70E-18	****	Wilcoxon
p__Firmicutes;c__Negativicutes;o__Selenomonadales;f__Veillonellaceae;g__Veillonella	FluB	HRV	2.77E-25	2.50E-24	****	Wilcoxon
p__Firmicutes;c__Negativicutes;o__Selenomonadales;f__Veillonellaceae;g__Veillonella	FluB	Infection	0.049593	0.099	*	Wilcoxon
p__Firmicutes;c__Negativicutes;o__Selenomonadales;f__Veillonellaceae;g__Veillonella	RSV	HRV	0.345558	0.35	ns	Wilcoxon
p__Firmicutes;c__Negativicutes;o__Selenomonadales;f__Veillonellaceae;g__Veillonella	RSV	Infection	9.34E-24	7.50E-23	****	Wilcoxon
p__Firmicutes;c__Negativicutes;o__Selenomonadales;f__Veillonellaceae;g__Veillonella	HRV	Infection	5.56E-29	5.60E-28	****	Wilcoxon
p__Bacteroidetes;c__Bacteroidia;o__Bacteroidales;f__Prevotellaceae;g__Alloprevotella	FluA	FluB	1.11E-26	1.00E-25	****	Wilcoxon
p__Bacteroidetes;c__Bacteroidia;o__Bacteroidales;f__Prevotellaceae;g__Alloprevotella	FluA	RSV	8.13E-09	2.40E-08	****	Wilcoxon
p__Bacteroidetes;c__Bacteroidia;o__Bacteroidales;f__Prevotellaceae;g__Alloprevotella	FluA	HRV	0.261253	0.26	ns	Wilcoxon
p__Bacteroidetes;c__Bacteroidia;o__Bacteroidales;f__Prevotellaceae;g__Alloprevotella	FluA	Infection	2.63E-18	1.80E-17	****	Wilcoxon
p__Bacteroidetes;c__Bacteroidia;o__Bacteroidales;f__Prevotellaceae;g__Alloprevotella	FluB	RSV	1.18E-14	7.10E-14	****	Wilcoxon
p__Bacteroidetes;c__Bacteroidia;o__Bacteroidales;f__Prevotellaceae;g__Alloprevotella	FluB	HRV	1.70E-31	1.70E-30	****	Wilcoxon
p__Bacteroidetes;c__Bacteroidia;o__Bacteroidales;f__Prevotellaceae;g__Alloprevotella	FluB	Infection	3.87E-11	1.50E-10	****	Wilcoxon
p__Bacteroidetes;c__Bacteroidia;o__Bacteroidales;f__Prevotellaceae;g__Alloprevotella	RSV	HRV	1.78E-08	3.60E-08	****	Wilcoxon
p__Bacteroidetes;c__Bacteroidia;o__Bacteroidales;f__Prevotellaceae;g__Alloprevotella	RSV	Infection	3.47E-14	1.70E-13	****	Wilcoxon
p__Bacteroidetes;c__Bacteroidia;o__Bacteroidales;f__Prevotellaceae;g__Alloprevotella	HRV	Infection	2.73E-23	2.20E-22	****	Wilcoxon
p__Actinobacteria;c__Actinobacteria;o__Actinomycetales;f__Actinomycetaceae;g__Actinomyces	FluA	FluB	4.40E-06	3.50E-05	****	Wilcoxon
p__Actinobacteria;c__Actinobacteria;o__Actinomycetales;f__Actinomycetaceae;g__Actinomyces	FluA	RSV	0.016368	0.082	*	Wilcoxon
p__Actinobacteria;c__Actinobacteria;o__Actinomycetales;f__Actinomycetaceae;g__Actinomyces	FluA	HRV	2.97E-07	2.70E-06	****	Wilcoxon
p__Actinobacteria;c__Actinobacteria;o__Actinomycetales;f__Actinomycetaceae;g__Actinomyces	FluA	Infection	4.77E-08	4.80E-07	****	Wilcoxon
p__Actinobacteria;c__Actinobacteria;o__Actinomycetales;f__Actinomycetaceae;g__Actinomyces	FluB	RSV	0.097467	0.29	ns	Wilcoxon
p__Actinobacteria;c__Actinobacteria;o__Actinomycetales;f__Actinomycetaceae;g__Actinomyces	FluB	HRV	0.000125	0.00075	***	Wilcoxon
p__Actinobacteria;c__Actinobacteria;o__Actinomycetales;f__Actinomycetaceae;g__Actinomyces	FluB	Infection	0.435704	0.87	ns	Wilcoxon
p__Actinobacteria;c__Actinobacteria;o__Actinomycetales;f__Actinomycetaceae;g__Actinomyces	RSV	HRV	0.481102	0.87	ns	Wilcoxon
p__Actinobacteria;c__Actinobacteria;o__Actinomycetales;f__Actinomycetaceae;g__Actinomyces	RSV	Infection	0.073494	0.29	ns	Wilcoxon
p__Actinobacteria;c__Actinobacteria;o__Actinomycetales;f__Actinomycetaceae;g__Actinomyces	HRV	Infection	3.13E-05	0.00022	****	Wilcoxon
p__Firmicutes;c__Erysipelotrichia;o__Erysipelotrichales;f__Erysipelotrichaceae;g__Solobacterium	FluA	FluB	7.78E-07	5.40E-06	****	Wilcoxon

p__Firmicutes;c__Erysipelotrichia;o__Erysipelotrichales;f__Erysipelotrichaceae;g__Solobacterium	FluA	RSV	3.05E-09	2.40E-08	****	Wilcoxon
p__Firmicutes;c__Erysipelotrichia;o__Erysipelotrichales;f__Erysipelotrichaceae;g__Solobacterium	FluA	HRV	1.63E-11	1.60E-10	****	Wilcoxon
p__Firmicutes;c__Erysipelotrichia;o__Erysipelotrichales;f__Erysipelotrichaceae;g__Solobacterium	FluA	Infection	1.42E-10	1.30E-09	****	Wilcoxon
p__Firmicutes;c__Erysipelotrichia;o__Erysipelotrichales;f__Erysipelotrichaceae;g__Solobacterium	FluB	RSV	0.383294	1	ns	Wilcoxon
p__Firmicutes;c__Erysipelotrichia;o__Erysipelotrichales;f__Erysipelotrichaceae;g__Solobacterium	FluB	HRV	0.004697	0.028	**	Wilcoxon
p__Firmicutes;c__Erysipelotrichia;o__Erysipelotrichales;f__Erysipelotrichaceae;g__Solobacterium	FluB	Infection	0.083102	0.42	ns	Wilcoxon
p__Firmicutes;c__Erysipelotrichia;o__Erysipelotrichales;f__Erysipelotrichaceae;g__Solobacterium	RSV	HRV	0.300664	1	ns	Wilcoxon
p__Firmicutes;c__Erysipelotrichia;o__Erysipelotrichales;f__Erysipelotrichaceae;g__Solobacterium	RSV	Infection	0.909065	1	ns	Wilcoxon
p__Firmicutes;c__Erysipelotrichia;o__Erysipelotrichales;f__Erysipelotrichaceae;g__Solobacterium	HRV	Infection	0.532642	1	ns	Wilcoxon
p__Proteobacteria;c__Gammaproteobacteria;o__Enterobacteriales;f__Enterobacteriaceae	FluA	FluB	0.066916	0.33	ns	Wilcoxon
p__Proteobacteria;c__Gammaproteobacteria;o__Enterobacteriales;f__Enterobacteriaceae	FluA	RSV	0.002375	0.024	**	Wilcoxon
p__Proteobacteria;c__Gammaproteobacteria;o__Enterobacteriales;f__Enterobacteriaceae	FluA	HRV	0.008529	0.06	**	Wilcoxon
p__Proteobacteria;c__Gammaproteobacteria;o__Enterobacteriales;f__Enterobacteriaceae	FluA	Infection	0.203285	0.81	ns	Wilcoxon
p__Proteobacteria;c__Gammaproteobacteria;o__Enterobacteriales;f__Enterobacteriaceae	FluB	RSV	0.003885	0.031	**	Wilcoxon
p__Proteobacteria;c__Gammaproteobacteria;o__Enterobacteriales;f__Enterobacteriaceae	FluB	HRV	0.002361	0.024	**	Wilcoxon
p__Proteobacteria;c__Gammaproteobacteria;o__Enterobacteriales;f__Enterobacteriaceae	FluB	Infection	0.312946	0.94	ns	Wilcoxon
p__Proteobacteria;c__Gammaproteobacteria;o__Enterobacteriales;f__Enterobacteriaceae	RSV	HRV	0.626976	1	ns	Wilcoxon
p__Proteobacteria;c__Gammaproteobacteria;o__Enterobacteriales;f__Enterobacteriaceae	RSV	Infection	0.021042	0.13	*	Wilcoxon
p__Proteobacteria;c__Gammaproteobacteria;o__Enterobacteriales;f__Enterobacteriaceae	HRV	Infection	0.792703	1	ns	Wilcoxon
p__Bacteroidetes;c__Bacteroidia;o__Bacteroidales;f__Tannerellaceae;g__Tannerella;s__uncultured bacterium	FluA	FluB	1.11E-26	1.00E-25	****	Wilcoxon
p__Bacteroidetes;c__Bacteroidia;o__Bacteroidales;f__Tannerellaceae;g__Tannerella;s__uncultured bacterium	FluA	RSV	8.13E-09	2.40E-08	****	Wilcoxon
p__Bacteroidetes;c__Bacteroidia;o__Bacteroidales;f__Tannerellaceae;g__Tannerella;s__uncultured bacterium	FluA	HRV	0.261253	0.26	ns	Wilcoxon
p__Bacteroidetes;c__Bacteroidia;o__Bacteroidales;f__Tannerellaceae;g__Tannerella;s__uncultured bacterium	FluA	Infection	2.63E-18	1.80E-17	****	Wilcoxon
p__Bacteroidetes;c__Bacteroidia;o__Bacteroidales;f__Tannerellaceae;g__Tannerella;s__uncultured bacterium	FluB	RSV	1.18E-14	7.10E-14	****	Wilcoxon
p__Bacteroidetes;c__Bacteroidia;o__Bacteroidales;f__Tannerellaceae;g__Tannerella;s__uncultured bacterium	FluB	HRV	1.70E-31	1.70E-30	****	Wilcoxon
p__Bacteroidetes;c__Bacteroidia;o__Bacteroidales;f__Tannerellaceae;g__Tannerella;s__uncultured bacterium	FluB	Infection	3.87E-11	1.50E-10	****	Wilcoxon
p__Bacteroidetes;c__Bacteroidia;o__Bacteroidales;f__Tannerellaceae;g__Tannerella;s__uncultured bacterium	RSV	HRV	1.78E-08	3.60E-08	****	Wilcoxon
p__Bacteroidetes;c__Bacteroidia;o__Bacteroidales;f__Tannerellaceae;g__Tannerella;s__uncultured bacterium	RSV	Infection	3.47E-14	1.70E-13	****	Wilcoxon
p__Bacteroidetes;c__Bacteroidia;o__Bacteroidales;f__Tannerellaceae;g__Tannerella;s__uncultured bacterium	HRV	Infection	2.73E-23	2.20E-22	****	Wilcoxon
p__Bacteroidetes;c__Bacteroidia;o__Bacteroidales;f__Prevotellaceae;g__Prevotella 2;s__Prevotella shahii DSM 15611 = JCM 12083	FluA	FluB	4.40E-06	3.50E-05	****	Wilcoxon
p__Bacteroidetes;c__Bacteroidia;o__Bacteroidales;f__Prevotellaceae;g__Prevotella 2;s__Prevotella shahii DSM 15611 = JCM 12083	FluA	RSV	0.016368	0.082	*	Wilcoxon
p__Bacteroidetes;c__Bacteroidia;o__Bacteroidales;f__Prevotellaceae;g__Prevotella 2;s__Prevotella shahii DSM 15611 = JCM 12083	FluA	HRV	2.97E-07	2.70E-06	****	Wilcoxon
p__Bacteroidetes;c__Bacteroidia;o__Bacteroidales;f__Prevotellaceae;g__Prevotella 2;s__Prevotella shahii DSM 15611 = JCM 12083	FluA	Infection	4.77E-08	4.80E-07	****	Wilcoxon
p__Bacteroidetes;c__Bacteroidia;o__Bacteroidales;f__Prevotellaceae;g__Prevotella 2;s__Prevotella shahii DSM 15611 = JCM 12083	FluB	RSV	0.097467	0.29	ns	Wilcoxon
p__Bacteroidetes;c__Bacteroidia;o__Bacteroidales;f__Prevotellaceae;g__Prevotella 2;s__Prevotella shahii DSM 15611 = JCM 12083	FluB	HRV	0.000125	0.00075	***	Wilcoxon
p__Bacteroidetes;c__Bacteroidia;o__Bacteroidales;f__Prevotellaceae;g__Prevotella 2;s__Prevotella shahii DSM 15611 = JCM 12083	FluB	Infection	0.435704	0.87	ns	Wilcoxon
p__Bacteroidetes;c__Bacteroidia;o__Bacteroidales;f__Prevotellaceae;g__Prevotella 2;s__Prevotella shahii DSM 15611 = JCM 12083	RSV	HRV	0.481102	0.87	ns	Wilcoxon

p__Bacteroidetes;c__Bacteroidia;o__Bacteroidales;f__Prevotellaceae;g__Prevotella 2;s__Prevotella shahii DSM 15611 = JCM 12083	RSV	Infection	0.073494	0.29	ns	Wilcoxon
p__Bacteroidetes;c__Bacteroidia;o__Bacteroidales;f__Prevotellaceae;g__Prevotella 2;s__Prevotella shahii DSM 15611 = JCM 12083	HRV	Infection	3.13E-05	0.00022	****	Wilcoxon
p__Firmicutes;c__Bacilli;o__Lactobacillales;f__Aerococcaceae;g__Abiotrophia;s__uncultured bacterium	FluA	FluB	1.11E-26	1.00E-25	****	Wilcoxon
p__Firmicutes;c__Bacilli;o__Lactobacillales;f__Aerococcaceae;g__Abiotrophia;s__uncultured bacterium	FluA	RSV	8.13E-09	2.40E-08	****	Wilcoxon
p__Firmicutes;c__Bacilli;o__Lactobacillales;f__Aerococcaceae;g__Abiotrophia;s__uncultured bacterium	FluA	HRV	0.261253	0.26	ns	Wilcoxon
p__Firmicutes;c__Bacilli;o__Lactobacillales;f__Aerococcaceae;g__Abiotrophia;s__uncultured bacterium	FluA	Infection	2.63E-18	1.80E-17	****	Wilcoxon
p__Firmicutes;c__Bacilli;o__Lactobacillales;f__Aerococcaceae;g__Abiotrophia;s__uncultured bacterium	FluB	RSV	1.18E-14	7.10E-14	****	Wilcoxon
p__Firmicutes;c__Bacilli;o__Lactobacillales;f__Aerococcaceae;g__Abiotrophia;s__uncultured bacterium	FluB	HRV	1.70E-31	1.70E-30	****	Wilcoxon
p__Firmicutes;c__Bacilli;o__Lactobacillales;f__Aerococcaceae;g__Abiotrophia;s__uncultured bacterium	FluB	Infection	3.87E-11	1.50E-10	****	Wilcoxon
p__Firmicutes;c__Bacilli;o__Lactobacillales;f__Aerococcaceae;g__Abiotrophia;s__uncultured bacterium	RSV	HRV	1.78E-08	3.60E-08	****	Wilcoxon
p__Firmicutes;c__Bacilli;o__Lactobacillales;f__Aerococcaceae;g__Abiotrophia;s__uncultured bacterium	RSV	Infection	3.47E-14	1.70E-13	****	Wilcoxon
p__Firmicutes;c__Bacilli;o__Lactobacillales;f__Aerococcaceae;g__Abiotrophia;s__uncultured bacterium	HRV	Infection	2.73E-23	2.20E-22	****	Wilcoxon
p__Firmicutes;c__Clostridia;o__Clostridiales;f__Peptostreptococcaceae;g__Peptostreptococcus;s__uncultured organism	FluA	FluB	7.78E-07	5.40E-06	****	Wilcoxon
p__Firmicutes;c__Clostridia;o__Clostridiales;f__Peptostreptococcaceae;g__Peptostreptococcus;s__uncultured organism	FluA	RSV	3.05E-09	2.40E-08	****	Wilcoxon
p__Firmicutes;c__Clostridia;o__Clostridiales;f__Peptostreptococcaceae;g__Peptostreptococcus;s__uncultured organism	FluA	HRV	1.63E-11	1.60E-10	****	Wilcoxon
p__Firmicutes;c__Clostridia;o__Clostridiales;f__Peptostreptococcaceae;g__Peptostreptococcus;s__uncultured organism	FluA	Infection	1.42E-10	1.30E-09	****	Wilcoxon
p__Firmicutes;c__Clostridia;o__Clostridiales;f__Peptostreptococcaceae;g__Peptostreptococcus;s__uncultured organism	FluB	RSV	0.383294	1	ns	Wilcoxon
p__Firmicutes;c__Clostridia;o__Clostridiales;f__Peptostreptococcaceae;g__Peptostreptococcus;s__uncultured organism	FluB	HRV	0.004697	0.028	**	Wilcoxon
p__Firmicutes;c__Clostridia;o__Clostridiales;f__Peptostreptococcaceae;g__Peptostreptococcus;s__uncultured organism	FluB	Infection	0.083102	0.42	ns	Wilcoxon
p__Firmicutes;c__Clostridia;o__Clostridiales;f__Peptostreptococcaceae;g__Peptostreptococcus;s__uncultured organism	RSV	HRV	0.300664	1	ns	Wilcoxon
p__Firmicutes;c__Clostridia;o__Clostridiales;f__Peptostreptococcaceae;g__Peptostreptococcus;s__uncultured organism	RSV	Infection	0.909065	1	ns	Wilcoxon
p__Firmicutes;c__Clostridia;o__Clostridiales;f__Peptostreptococcaceae;g__Peptostreptococcus;s__uncultured organism	HRV	Infection	0.532642	1	ns	Wilcoxon
p__Fusobacteria;c__Fusobacteriia;o__Fusobacteriales;f__Fusobacteriaceae;g__Fusobacterium	FluA	FluB	0.00175	0.0088	**	Wilcoxon
p__Fusobacteria;c__Fusobacteriia;o__Fusobacteriales;f__Fusobacteriaceae;g__Fusobacterium	FluA	HRV	0.021263	0.044	*	Wilcoxon
p__Fusobacteria;c__Fusobacteriia;o__Fusobacteriales;f__Fusobacteriaceae;g__Fusobacterium	FluA	Infection	7.23E-05	0.00043	****	Wilcoxon
p__Fusobacteria;c__Fusobacteriia;o__Fusobacteriales;f__Fusobacteriaceae;g__Fusobacterium	FluB	HRV	0.266667	0.27	ns	Wilcoxon
p__Fusobacteria;c__Fusobacteriia;o__Fusobacteriales;f__Fusobacteriaceae;g__Fusobacterium	FluB	Infection	0.003612	0.014	**	Wilcoxon
p__Fusobacteria;c__Fusobacteriia;o__Fusobacteriales;f__Fusobacteriaceae;g__Fusobacterium	HRV	Infection	0.014706	0.044	*	Wilcoxon
p__Bacteroidetes;c__Bacteroidia;o__Bacteroidales;f__Prevotellaceae;g__Alloprevotella;s__uncultured Prevotellaceae bacterium	FluA	FluB	5.23E-08	2.60E-07	****	Wilcoxon
p__Bacteroidetes;c__Bacteroidia;o__Bacteroidales;f__Prevotellaceae;g__Alloprevotella;s__uncultured Prevotellaceae bacterium	FluA	RSV	3.17E-07	1.30E-06	****	Wilcoxon
p__Bacteroidetes;c__Bacteroidia;o__Bacteroidales;f__Prevotellaceae;g__Alloprevotella;s__uncultured Prevotellaceae bacterium	FluA	HRV	7.28E-12	5.80E-11	****	Wilcoxon
p__Bacteroidetes;c__Bacteroidia;o__Bacteroidales;f__Prevotellaceae;g__Alloprevotella;s__uncultured Prevotellaceae bacterium	FluA	Infection	0.014253	0.029	*	Wilcoxon
p__Bacteroidetes;c__Bacteroidia;o__Bacteroidales;f__Prevotellaceae;g__Alloprevotella;s__uncultured Prevotellaceae bacterium	FluB	RSV	1.74E-09	1.20E-08	****	Wilcoxon
p__Bacteroidetes;c__Bacteroidia;o__Bacteroidales;f__Prevotellaceae;g__Alloprevotella;s__uncultured Prevotellaceae bacterium	FluB	HRV	6.18E-23	6.20E-22	****	Wilcoxon
p__Bacteroidetes;c__Bacteroidia;o__Bacteroidales;f__Prevotellaceae;g__Alloprevotella;s__uncultured Prevotellaceae bacterium	FluB	Infection	0.002755	0.0083	**	Wilcoxon
p__Bacteroidetes;c__Bacteroidia;o__Bacteroidales;f__Prevotellaceae;g__Alloprevotella;s__uncultured Prevotellaceae bacterium	RSV	HRV	0.159414	0.16	ns	Wilcoxon
p__Bacteroidetes;c__Bacteroidia;o__Bacteroidales;f__Prevotellaceae;g__Alloprevotella;s__uncultured Prevotellaceae bacterium	RSV	Infection	2.46E-08	1.50E-07	****	Wilcoxon

p__Bacteroidetes;c__Bacteroidia;o__Bacteroidales;f__Prevotellaceae;g__Alloprevotella;s__uncultured Prevotellaceae bacterium	HRV	Infection	1.53E-16	1.40E-15	****	Wilcoxon
p__Firmicutes;c__Clostridia;o__Clostridiales;f__Peptostreptococcaceae;g__[Eubacterium] yurii group;s__uncultured bacterium	FluA	FluB	3.14E-17	2.80E-16	****	Wilcoxon
p__Firmicutes;c__Clostridia;o__Clostridiales;f__Peptostreptococcaceae;g__[Eubacterium] yurii group;s__uncultured bacterium	FluA	RSV	0.002479	0.0074	**	Wilcoxon
p__Firmicutes;c__Clostridia;o__Clostridiales;f__Peptostreptococcaceae;g__[Eubacterium] yurii group;s__uncultured bacterium	FluA	HRV	0.013809	0.028	*	Wilcoxon
p__Firmicutes;c__Clostridia;o__Clostridiales;f__Peptostreptococcaceae;g__[Eubacterium] yurii group;s__uncultured bacterium	FluA	Infection	4.25E-09	3.00E-08	****	Wilcoxon
p__Firmicutes;c__Clostridia;o__Clostridiales;f__Peptostreptococcaceae;g__[Eubacterium] yurii group;s__uncultured bacterium	FluB	RSV	2.01E-11	1.60E-10	****	Wilcoxon
p__Firmicutes;c__Clostridia;o__Clostridiales;f__Peptostreptococcaceae;g__[Eubacterium] yurii group;s__uncultured bacterium	FluB	HRV	2.15E-18	2.20E-17	****	Wilcoxon
p__Firmicutes;c__Clostridia;o__Clostridiales;f__Peptostreptococcaceae;g__[Eubacterium] yurii group;s__uncultured bacterium	FluB	Infection	0.000187	0.00093	***	Wilcoxon
p__Firmicutes;c__Clostridia;o__Clostridiales;f__Peptostreptococcaceae;g__[Eubacterium] yurii group;s__uncultured bacterium	RSV	HRV	0.138501	0.14	ns	Wilcoxon
p__Firmicutes;c__Clostridia;o__Clostridiales;f__Peptostreptococcaceae;g__[Eubacterium] yurii group;s__uncultured bacterium	RSV	Infection	0.001067	0.0043	**	Wilcoxon
p__Firmicutes;c__Clostridia;o__Clostridiales;f__Peptostreptococcaceae;g__[Eubacterium] yurii group;s__uncultured bacterium	HRV	Infection	3.64E-07	2.20E-06	****	Wilcoxon
p__Patescibacteria;c__Gracilibacteria;o__Absconditabacteriales (SR1);f__SR1 bacterium oral taxon 875;g__SR1 bacterium oral taxon 875;s__SR1 bacterium oral taxon 875	FluA	FluB	1.40E-05	9.80E-05	****	Wilcoxon
p__Patescibacteria;c__Gracilibacteria;o__Absconditabacteriales (SR1);f__SR1 bacterium oral taxon 875;g__SR1 bacterium oral taxon 875;s__SR1 bacterium oral taxon 875	FluA	RSV	9.85E-06	7.90E-05	****	Wilcoxon
p__Patescibacteria;c__Gracilibacteria;o__Absconditabacteriales (SR1);f__SR1 bacterium oral taxon 875;g__SR1 bacterium oral taxon 875;s__SR1 bacterium oral taxon 875	FluA	HRV	1.53E-05	9.80E-05	****	Wilcoxon
p__Patescibacteria;c__Gracilibacteria;o__Absconditabacteriales (SR1);f__SR1 bacterium oral taxon 875;g__SR1 bacterium oral taxon 875;s__SR1 bacterium oral taxon 875	FluA	Infection	1.59E-05	9.80E-05	****	Wilcoxon
p__Patescibacteria;c__Gracilibacteria;o__Absconditabacteriales (SR1);f__SR1 bacterium oral taxon 875;g__SR1 bacterium oral taxon 875;s__SR1 bacterium oral taxon 875	FluB	RSV	0.098959	0.3	ns	Wilcoxon
p__Patescibacteria;c__Gracilibacteria;o__Absconditabacteriales (SR1);f__SR1 bacterium oral taxon 875;g__SR1 bacterium oral taxon 875;s__SR1 bacterium oral taxon 875	FluB	HRV	0.653747	0.65	ns	Wilcoxon
p__Patescibacteria;c__Gracilibacteria;o__Absconditabacteriales (SR1);f__SR1 bacterium oral taxon 875;g__SR1 bacterium oral taxon 875;s__SR1 bacterium oral taxon 875	FluB	Infection	5.45E-08	5.50E-07	****	Wilcoxon
p__Patescibacteria;c__Gracilibacteria;o__Absconditabacteriales (SR1);f__SR1 bacterium oral taxon 875;g__SR1 bacterium oral taxon 875;s__SR1 bacterium oral taxon 875	RSV	HRV	0.219807	0.44	ns	Wilcoxon
p__Patescibacteria;c__Gracilibacteria;o__Absconditabacteriales (SR1);f__SR1 bacterium oral taxon 875;g__SR1 bacterium oral taxon 875;s__SR1 bacterium oral taxon 875	RSV	Infection	2.03E-05	9.80E-05	****	Wilcoxon
p__Patescibacteria;c__Gracilibacteria;o__Absconditabacteriales (SR1);f__SR1 bacterium oral taxon 875;g__SR1 bacterium oral taxon 875;s__SR1 bacterium oral taxon 875	HRV	Infection	6.27E-07	5.60E-06	****	Wilcoxon
p__Fusobacteria;c__Fusobacteriia;o__Fusobacteriales;f__Leptotrichiaceae;g__Leptotrichia;s__Leptotrichia sp. oral clone FP036	FluA	FluB	5.23E-08	2.60E-07	****	Wilcoxon
p__Fusobacteria;c__Fusobacteriia;o__Fusobacteriales;f__Leptotrichiaceae;g__Leptotrichia;s__Leptotrichia sp. oral clone FP036	FluA	RSV	3.17E-07	1.30E-06	****	Wilcoxon
p__Fusobacteria;c__Fusobacteriia;o__Fusobacteriales;f__Leptotrichiaceae;g__Leptotrichia;s__Leptotrichia sp. oral clone FP036	FluA	HRV	7.28E-12	5.80E-11	****	Wilcoxon
p__Fusobacteria;c__Fusobacteriia;o__Fusobacteriales;f__Leptotrichiaceae;g__Leptotrichia;s__Leptotrichia sp. oral clone FP036	FluA	Infection	0.014253	0.029	*	Wilcoxon
p__Fusobacteria;c__Fusobacteriia;o__Fusobacteriales;f__Leptotrichiaceae;g__Leptotrichia;s__Leptotrichia sp. oral clone FP036	FluB	RSV	1.74E-09	1.20E-08	****	Wilcoxon
p__Fusobacteria;c__Fusobacteriia;o__Fusobacteriales;f__Leptotrichiaceae;g__Leptotrichia;s__Leptotrichia sp. oral clone FP036	FluB	HRV	6.18E-23	6.20E-22	****	Wilcoxon
p__Fusobacteria;c__Fusobacteriia;o__Fusobacteriales;f__Leptotrichiaceae;g__Leptotrichia;s__Leptotrichia sp. oral clone FP036	FluB	Infection	0.002755	0.0083	**	Wilcoxon
p__Fusobacteria;c__Fusobacteriia;o__Fusobacteriales;f__Leptotrichiaceae;g__Leptotrichia;s__Leptotrichia sp. oral clone FP036	RSV	HRV	0.159414	0.16	ns	Wilcoxon
p__Fusobacteria;c__Fusobacteriia;o__Fusobacteriales;f__Leptotrichiaceae;g__Leptotrichia;s__Leptotrichia sp. oral clone FP036	RSV	Infection	2.46E-08	1.50E-07	****	Wilcoxon
p__Fusobacteria;c__Fusobacteriia;o__Fusobacteriales;f__Leptotrichiaceae;g__Leptotrichia;s__Leptotrichia sp. oral clone FP036	HRV	Infection	1.53E-16	1.40E-15	****	Wilcoxon
p__Firmicutes;c__Clostridia;o__Clostridiales;f__Lachnospiraceae;g__Oribacterium;s__Oribacterium sinus	FluA	FluB	7.78E-07	5.40E-06	****	Wilcoxon
p__Firmicutes;c__Clostridia;o__Clostridiales;f__Lachnospiraceae;g__Oribacterium;s__Oribacterium sinus	FluA	RSV	3.05E-09	2.40E-08	****	Wilcoxon
p__Firmicutes;c__Clostridia;o__Clostridiales;f__Lachnospiraceae;g__Oribacterium;s__Oribacterium sinus	FluA	HRV	1.63E-11	1.60E-10	****	Wilcoxon
p__Firmicutes;c__Clostridia;o__Clostridiales;f__Lachnospiraceae;g__Oribacterium;s__Oribacterium sinus	FluA	Infection	1.42E-10	1.30E-09	****	Wilcoxon
p__Firmicutes;c__Clostridia;o__Clostridiales;f__Lachnospiraceae;g__Oribacterium;s__Oribacterium sinus	FluB	RSV	0.383294	1	ns	Wilcoxon
p__Firmicutes;c__Clostridia;o__Clostridiales;f__Lachnospiraceae;g__Oribacterium;s__Oribacterium sinus	FluB	HRV	0.004697	0.028	**	Wilcoxon

p__Firmicutes;c__Clostridia;o__Clostridiales;f__Lachnospiraceae;g__Oribacterium;s__Oribacterium sinus	FluB	Infection	0.083102	0.42	ns	Wilcoxon
p__Firmicutes;c__Clostridia;o__Clostridiales;f__Lachnospiraceae;g__Oribacterium;s__Oribacterium sinus	RSV	HRV	0.300664	1	ns	Wilcoxon
p__Firmicutes;c__Clostridia;o__Clostridiales;f__Lachnospiraceae;g__Oribacterium;s__Oribacterium sinus	RSV	Infection	0.909065	1	ns	Wilcoxon
p__Firmicutes;c__Clostridia;o__Clostridiales;f__Lachnospiraceae;g__Oribacterium;s__Oribacterium sinus	HRV	Infection	0.532642	1	ns	Wilcoxon
p__Firmicutes;c__Clostridia;o__Clostridiales;f__Lachnospiraceae;g__Oribacterium;s__Oribacterium parvum ACB1	FluA	FluB	0.066916	0.33	ns	Wilcoxon
p__Firmicutes;c__Clostridia;o__Clostridiales;f__Lachnospiraceae;g__Oribacterium;s__Oribacterium parvum ACB1	FluA	RSV	0.002375	0.024	**	Wilcoxon
p__Firmicutes;c__Clostridia;o__Clostridiales;f__Lachnospiraceae;g__Oribacterium;s__Oribacterium parvum ACB1	FluA	HRV	0.008529	0.06	**	Wilcoxon
p__Firmicutes;c__Clostridia;o__Clostridiales;f__Lachnospiraceae;g__Oribacterium;s__Oribacterium parvum ACB1	FluA	Infection	0.203285	0.81	ns	Wilcoxon
p__Firmicutes;c__Clostridia;o__Clostridiales;f__Lachnospiraceae;g__Oribacterium;s__Oribacterium parvum ACB1	FluB	RSV	0.003885	0.031	**	Wilcoxon
p__Firmicutes;c__Clostridia;o__Clostridiales;f__Lachnospiraceae;g__Oribacterium;s__Oribacterium parvum ACB1	FluB	HRV	0.002361	0.024	**	Wilcoxon
p__Firmicutes;c__Clostridia;o__Clostridiales;f__Lachnospiraceae;g__Oribacterium;s__Oribacterium parvum ACB1	FluB	Infection	0.312946	0.94	ns	Wilcoxon
p__Firmicutes;c__Clostridia;o__Clostridiales;f__Lachnospiraceae;g__Oribacterium;s__Oribacterium parvum ACB1	RSV	HRV	0.626976	1	ns	Wilcoxon
p__Firmicutes;c__Clostridia;o__Clostridiales;f__Lachnospiraceae;g__Oribacterium;s__Oribacterium parvum ACB1	RSV	Infection	0.021042	0.13	*	Wilcoxon
p__Firmicutes;c__Clostridia;o__Clostridiales;f__Lachnospiraceae;g__Oribacterium;s__Oribacterium parvum ACB1	HRV	Infection	0.792703	1	ns	Wilcoxon

Table S4. The Spearman interaction of differential pathways, differential ARGs, ARGs count, and differential species in the FluA group and healthy controls.

**Objects (A & B means A interacts with B)**	***R* *value***
Veillonella unclassified & PWY-5840: superpathway of menaquinol-7 biosynthesis	0.775
Veillonella unclassified & PWY-5899: superpathway of menaquinol-13 biosynthesis	0.771
Veillonella unclassified & PWY-5898: superpathway of menaquinol-12 biosynthesis	0.771
Veillonella unclassified & PWY-5897: superpathway of menaquinol-11 biosynthesis	0.771
Veillonella unclassified & PWY-5837: 1,4-dihydroxy-2-naphthoate biosynthesis I	0.782
Veillonella unclassified & PWY-5791: 1,4-dihydroxy-2-naphthoate biosynthesis II (plants)	0.782
Veillonella unclassified & PWY-5005: biotin biosynthesis II	0.833
Veillonella parvula & PWY-5005: biotin biosynthesis II	0.773
PWY-6897: thiamin salvage II & *Streptococcus constellatus*	0.660
PWY-6897: thiamin salvage II & *Rothia mucilaginosa*	-0.502
COA-PWY: coenzyme A biosynthesis I & *Veillonella parvula*	0.504
COA-PWY: coenzyme A biosynthesis I & *Rothia mucilaginosa*	-0.555
ARO_3003479 *tetR* & PWY-5840: superpathway of menaquinol-7 biosynthesis	-0.776
ARO_3003479 *tetR* & ARG+POLYAMINE-SYN: superpathway of arginine and polyamine biosynthesis	-0.760
ARO_3003479 *tetR* & ARGs count	0.714
ARO_3003479 *tetR* & Veillonella unclassified	-0.723
ARO_3003479 *tetR* & Rothia mucilaginosa	0.613
ARO_3000165 *tet(A)* & ARG+POLYAMINE-SYN: superpathway of arginine and polyamine biosynthesis	-0.776
ARO_3000165 *tet(A)* & ARGs count	0.703
ARO_3000165 *tet(A)* & *Veillonella unclassified*	-0.738
ARO_3000165 *tet(A)* & *Rothia mucilaginosa*	0.576
PWY-6147: 6-hydroxymethyl-dihydropterin diphosphate biosynthesis I & *Streptococcus constellatus*	0.589
PWY-6147: 6-hydroxymethyl-dihydropterin diphosphate biosynthesis I & *Rothia mucilaginosa*	-0.629
PWY-6147: 6-hydroxymethyl-dihydropterin diphosphate biosynthesis I & *Lachnospiraceae oral taxon 107*	0.508
SULFATE-CYS-PWY: superpathway of sulfate assimilation and cysteine biosynthesis & *Lachnospiraceae oral taxon 107*	0.659
PWY-7282: 4-amino-2-methyl-5-phosphomethylpyrimidine biosynthesis (yeast) & *Rothia mucilaginosa*	-0.544

PWY-7282: 4-amino-2-methyl-5-phosphomethylpyrimidine biosynthesis (yeast) & *Lachnospiraceae oral taxon 107*	0.682
PWY-5154: L-arginine biosynthesis III (via N-acetyl-L-citrulline) & ARO_3000194 *tetW*	0.473
PWY-5154: L-arginine biosynthesis III (via N-acetyl-L-citrulline) & *Veillonella unclassified*	0.626
PWY-5154: L-arginine biosynthesis III (via N-acetyl-L-citrulline) & *Veillonella parvula*	0.523
PWY-5154: L-arginine biosynthesis III (via N-acetyl-L-citrulline) & *Rothia mucilaginosa*	-0.594
PWY-5154: L-arginine biosynthesis III (via N-acetyl-L-citrulline) & *Lachnospiraceae oral taxon 107*	0.464
PANTOSYN-PWY: pantothenate and coenzyme A biosynthesis I & *Veillonella unclassified*	0.574
PANTOSYN-PWY: pantothenate and coenzyme A biosynthesis I & *Rothia mucilaginosa*	-0.630
PANTOSYN-PWY: pantothenate and coenzyme A biosynthesis I & *Lachnospiraceae oral taxon 107*	0.598
PANTOSYN-PWY: pantothenate and coenzyme A biosynthesis I & *Atopobium rimae*	0.648
P42-PWY: incomplete reductive TCA cycle & *Veillonella unclassified*	0.447
P42-PWY: incomplete reductive TCA cycle & *Lachnospiraceae oral taxon 107*	0.585
P42-PWY: incomplete reductive TCA cycle & *Atopobium rimae*	0.600
POLYAMSYN-PWY: superpathway of polyamine biosynthesis I & ARO_3003479 *tetR*	-0.729
POLYAMSYN-PWY: superpathway of polyamine biosynthesis I & ARO_3000165 *tet(A)*	-0.738
POLYAMSYN-PWY: superpathway of polyamine biosynthesis I & ARO_3000194 *tetW*	0.458
POLYAMSYN-PWY: superpathway of polyamine biosynthesis I & ARGs count	-0.615
POLYAMSYN-PWY: superpathway of polyamine biosynthesis I & *Veillonella unclassified*	0.651
POLYAMSYN-PWY: superpathway of polyamine biosynthesis I & *Rothia mucilaginosa*	-0.528
POLYAMSYN-PWY: superpathway of polyamine biosynthesis I & *Atopobium rimae*	0.471
PWY-5840: superpathway of menaquinol-7 biosynthesis & ARO_3003479 *tetR*	-0.776
PWY-5840: superpathway of menaquinol-7 biosynthesis & ARO_3000165 *tet(A)*	-0.756
PWY-5840: superpathway of menaquinol-7 biosynthesis & ARGs count	-0.747
PWY-5840: superpathway of menaquinol-7 biosynthesis & *Veillonella unclassified*	0.775
PWY-5840: superpathway of menaquinol-7 biosynthesis & *Veillonella parvula*	0.588

PWY-5840: superpathway of menaquinol-7 biosynthesis & *Rothia mucilaginosa*	-0.669
PWY-5840: superpathway of menaquinol-7 biosynthesis & *Atopobium rimae*	0.644
PWY-4242: pantothenate and coenzyme A biosynthesis III & *Veillonella unclassified*	0.534
PWY-4242: pantothenate and coenzyme A biosynthesis III & *Veillonella parvula*	0.568
PWY-4242: pantothenate and coenzyme A biosynthesis III & *Rothia mucilaginosa*	-0.711
PWY-4242: pantothenate and coenzyme A biosynthesis III & *Lachnospiraceae oral taxon 107*	0.514
PWY-4242: pantothenate and coenzyme A biosynthesis III & *Atopobium rimae*	0.616
PWY-5899: superpathway of menaquinol-13 biosynthesis & ARO_3003479 *tetR*	-0.510
PWY-5899: superpathway of menaquinol-13 biosynthesis & ARO_3000165 *tet(A)*	-0.543
PWY-5899: superpathway of menaquinol-13 biosynthesis & *Veillonella unclassified*	0.771
PWY-5899: superpathway of menaquinol-13 biosynthesis & *Veillonella parvula*	0.665
PWY-5899: superpathway of menaquinol-13 biosynthesis & *Rothia mucilaginosa*	-0.710
PWY-5899: superpathway of menaquinol-13 biosynthesis & *Lachnospiraceae oral taxon 107*	0.559
PWY-5899: superpathway of menaquinol-13 biosynthesis & *Atopobium rimae*	0.681
PWY-5898: superpathway of menaquinol-12 biosynthesis & ARO_3003479 *tetR*	-0.510
PWY-5898: superpathway of menaquinol-12 biosynthesis & ARO_3000165 *tet(A)*	-0.543
PWY-5898: superpathway of menaquinol-12 biosynthesis & *Veillonella unclassified*	0.771
PWY-5898: superpathway of menaquinol-12 biosynthesis & *Veillonella parvula*	0.665
PWY-5898: superpathway of menaquinol-12 biosynthesis & *Rothia mucilaginosa*	-0.710
PWY-5898: superpathway of menaquinol-12 biosynthesis & *Lachnospiraceae oral taxon 107*	0.559
PWY-5898: superpathway of menaquinol-12 biosynthesis & *Atopobium rimae*	0.681
PWY-5897: superpathway of menaquinol-11 biosynthesis & ARO_3003479 *tetR*	-0.510
PWY-5897: superpathway of menaquinol-11 biosynthesis & ARO_3000165 *tet(A)*	-0.543
PWY-5897: superpathway of menaquinol-11 biosynthesis & *Veillonella unclassified*	0.771
PWY-5897: superpathway of menaquinol-11 biosynthesis & *Veillonella parvula*	0.665
PWY-5897: superpathway of menaquinol-11 biosynthesis & *Rothia mucilaginosa*	-0.710
PWY-5897: superpathway of menaquinol-11 biosynthesis & *Lachnospiraceae oral taxon 107*	0.559
PWY-5897: superpathway of menaquinol-11 biosynthesis & *Atopobium rimae*	0.681
ARG+POLYAMINE-SYN: superpathway of arginine and polyamine biosynthesis & ARO_3003479 *tetR*	-0.760
ARG+POLYAMINE-SYN: superpathway of arginine and polyamine biosynthesis & ARO_3000165 *tet(A)*	-0.776
ARG+POLYAMINE-SYN: superpathway of arginine and polyamine biosynthesis & ARO_3000194 *tetW*	0.507

ARG+POLYAMINE-SYN: superpathway of arginine and polyamine biosynthesis & ARGs count	-0.611
ARG+POLYAMINE-SYN: superpathway of arginine and polyamine biosynthesis & *Veillonella unclassified*	0.720
ARG+POLYAMINE-SYN: superpathway of arginine and polyamine biosynthesis & *Rothia mucilaginosa*	-0.553
ARG+POLYAMINE-SYN: superpathway of arginine and polyamine biosynthesis & *Atopobium rimae*	0.513
PWY0-1586: peptidoglycan maturation (meso-diaminopimelate containing) & ARO_3003479 *tetR*	-0.556
PWY0-1586: peptidoglycan maturation (meso-diaminopimelate containing) & ARO_3000165 *tet(A)*	-0.559
PWY0-1586: peptidoglycan maturation (meso-diaminopimelate containing) & *Veillonella unclassified*	0.661
PWY0-1586: peptidoglycan maturation (meso-diaminopimelate containing) & *Veillonella parvula*	0.660
PWY0-1586: peptidoglycan maturation (meso-diaminopimelate containing) & *Rothia mucilaginosa*	-0.701
PWY0-1586: peptidoglycan maturation (meso-diaminopimelate containing) & *Lachnospiraceae oral taxon 107*	0.518
PWY0-1586: peptidoglycan maturation (meso-diaminopimelate containing) & *Atopobium rimae*	0.648
PWY-5837: 1,4-dihydroxy-2-naphthoate biosynthesis I & ARO_3003479 *tetR*	-0.504
PWY-5837: 1,4-dihydroxy-2-naphthoate biosynthesis I & ARO_3000165 *tet(A)*	-0.547
PWY-5837: 1,4-dihydroxy-2-naphthoate biosynthesis I & ARO_3000194 *tetW*	0.506
PWY-5837: 1,4-dihydroxy-2-naphthoate biosynthesis I & ARGs count	-0.474
PWY-5837: 1,4-dihydroxy-2-naphthoate biosynthesis I & *Veillonella unclassified*	0.782
PWY-5837: 1,4-dihydroxy-2-naphthoate biosynthesis I & *Veillonella parvula*	0.727
PWY-5837: 1,4-dihydroxy-2-naphthoate biosynthesis I & *Rothia mucilaginosa*	-0.703
PWY-5837: 1,4-dihydroxy-2-naphthoate biosynthesis I & *Atopobium rimae*	0.582
PWY-5791: 1,4-dihydroxy-2-naphthoate biosynthesis II (plants) & ARO_3003479 *tetR*	-0.504
PWY-5791: 1,4-dihydroxy-2-naphthoate biosynthesis II (plants) & ARO_3000165 *tet(A)*	-0.547
PWY-5791: 1,4-dihydroxy-2-naphthoate biosynthesis II (plants) & ARO_3000194 *tetW*	0.506
PWY-5791: 1,4-dihydroxy-2-naphthoate biosynthesis II (plants) & ARGs count	-0.474
PWY-5791: 1,4-dihydroxy-2-naphthoate biosynthesis II (plants) & *Veillonella unclassified*	0.782
PWY-5791: 1,4-dihydroxy-2-naphthoate biosynthesis II (plants) & *Veillonella parvula*	0.727
PWY-5791: 1,4-dihydroxy-2-naphthoate biosynthesis II (plants) & *Rothia mucilaginosa*	-0.703

PWY-5791: 1,4-dihydroxy-2-naphthoate biosynthesis II (plants) & *Atopobium rimae*	0.582
PWY-5005: biotin biosynthesis II & ARO_3003479 *tetR*	-0.555
PWY-5005: biotin biosynthesis II & ARO_3000165 *tet(A)*	-0.590
PWY-5005: biotin biosynthesis II & ARO_3000194 *tetW*	0.468
PWY-5005: biotin biosynthesis II & ARGs count	-0.575
PWY-5005: biotin biosynthesis II & *Veillonella unclassified*	0.833
PWY-5005: biotin biosynthesis II & *Veillonella parvula*	0.773
PWY-5005: biotin biosynthesis II & *Rothia mucilaginosa*	-0.594
PWY-5005: biotin biosynthesis II & *Atopobium rimae*	0.572
ARO_3000194 *tetW* & ARGs count	-0.494
ARO_3000194 *tetW* & Veillonella unclassified	0.644
ARO_3000194 *tetW* & Aggregatibacter unclassified	-0.517
ARGs count & *Veillonella unclassified*	-0.703
ARGs count & *Gemella morbillorum*	0.473
ARGs count & *Atopobium rimae*	-0.459
ARGs count & *Aggregatibacter unclassified*	0.449

Table S5. The growth rate of potential pathogens in situ calculated by GRID algorithm

Potential pathogens	Growth rate	Coverage	Groups
Fusobacterium nucleatum	2.06	3.33	FluB
Fusobacterium nucleatum	2.04	4.28	FluA/FluB
Fusobacterium nucleatum	1.84	20.81	FluB
Fusobacterium nucleatum	1.44	13.78	FluB
Fusobacterium nucleatum	1.75	0.99	FluA
Fusobacterium nucleatum	1.73	10.53	FluA
Fusobacterium nucleatum	1.46	1.49	FluA
Fusobacterium nucleatum	1.7	4.70	FluB
Fusobacterium nucleatum	1.37	0.42	FluA/FluB
Fusobacterium nucleatum	1.54	0.28	FluA
Fusobacterium nucleatum	1.48	0.26	FluA
Fusobacterium nucleatum	1.79	0.24	FluA
Fusobacterium nucleatum	1.86	19.31	FluA/FluB
Fusobacterium nucleatum	1.59	1.60	FluA
Fusobacterium nucleatum	1	0.27	FluB
Fusobacterium nucleatum	1.45	1.18	FluA/FluB
Fusobacterium nucleatum	1.62	2.41	FluA
Fusobacterium nucleatum	1.81	1.36	FluA/FluB
Fusobacterium nucleatum	2.27	1.85	FluA/FluB
Fusobacterium nucleatum	1.98	4.82	FluA/FluB
Fusobacterium nucleatum	2.03	6.65	FluA
Fusobacterium nucleatum	2.01	2.41	FluA/FluB
Fusobacterium nucleatum	2.36	0.90	FluA/FluB
Fusobacterium nucleatum	1.72	1.23	FluA
Fusobacterium nucleatum	1.66	2.65	FluA/FluB
Fusobacterium nucleatum	2.01	6.18	FluB
Fusobacterium nucleatum	1.63	0.28	FluB
Fusobacterium nucleatum	1.63	0.77	FluB
Fusobacterium nucleatum	1.63	20.04	FluB
Fusobacterium nucleatum	1.6	1.17	Health
Fusobacterium nucleatum	2.21	1.44	Health
Fusobacterium nucleatum	1.46	0.24	Health
Fusobacterium nucleatum	2.08	2.17	Health
Fusobacterium nucleatum	1.38	0.57	Health
Fusobacterium nucleatum	1.57	1.16	Health
Fusobacterium nucleatum	1.7	0.70	Health
Fusobacterium nucleatum	1.63	0.28	Health
Fusobacterium nucleatum	1.45	1.09	Health
Fusobacterium nucleatum	1.59	0.61	Health
Haemophilus influenzae	1	0.23	FluA/FluB
Haemophilus influenzae	1.45	1.18	FluB

Haemophilus influenzae	1.31	1.05	FluB
Haemophilus influenzae	1.24	0.24	FluB
Haemophilus influenzae	1.53	0.82	FluA
Haemophilus influenzae	1.47	0.42	FluA
Haemophilus influenzae	1.12	0.91	FluB
Haemophilus influenzae	1.55	0.35	FluA/FluB
Haemophilus influenzae	1.35	1.64	FluA
Haemophilus influenzae	1	0.37	FluB
Haemophilus influenzae	1	0.36	FluA/FluB
Haemophilus influenzae	1.69	5.66	FluA/FluB
Haemophilus influenzae	1	0.42	FluA/FluB
Haemophilus influenzae	1.21	1.64	FluA
Haemophilus influenzae	1.27	3.55	FluA/FluB
Haemophilus influenzae	1.31	0.51	FluA/FluB
Haemophilus influenzae	1.53	0.98	FluA/FluB
Haemophilus influenzae	1.7	0.81	FluB
Haemophilus influenzae	1.19	1.01	FluB
Haemophilus influenzae	1.5	0.29	Health
Haemophilus influenzae	1.22	0.34	Health
Haemophilus influenzae	1.26	0.87	Health
Haemophilus influenzae	1.13	1.09	Health
Haemophilus influenzae	1.2	0.48	Health
Haemophilus influenzae	1	0.21	Health
Haemophilus influenzae	1	0.22	Health
Haemophilus sputorum	1	0.53	FluB
Haemophilus sputorum	2.36	1.19	FluA/FluB
Haemophilus sputorum	8.61	3.33	FluB
Haemophilus sputorum	2.2	6.01	FluB
Haemophilus sputorum	1	0.21	FluB
Haemophilus sputorum	2.32	6.78	FluA
Haemophilus sputorum	2.44	1.89	FluA
Haemophilus sputorum	3.01	2.72	FluB
Haemophilus sputorum	2.09	0.41	FluA/FluB
Haemophilus sputorum	3.82	1.07	FluA
Haemophilus sputorum	2.04	0.38	FluA
Haemophilus sputorum	1	0.54	FluB
Haemophilus sputorum	2.04	1.95	FluA/FluB
Haemophilus sputorum	2.47	0.42	FluA
Haemophilus sputorum	2.31	1.92	FluA/FluB
Haemophilus sputorum	3.04	1.30	FluA/FluB
Haemophilus sputorum	1	1.45	FluA/FluB
Haemophilus sputorum	2.39	4.24	FluA/FluB
Haemophilus sputorum	1	0.29	FluA/FluB
Haemophilus sputorum	3.55	0.46	FluA

Haemophilus sputorum	4.55	1.03	FluA/FluB
Haemophilus sputorum	1	0.95	FluB
Haemophilus sputorum	1	0.26	FluB
Haemophilus sputorum	1	0.20	FluB
Haemophilus sputorum	3.36	0.47	FluB
Haemophilus sputorum	1	0.48	Health
Haemophilus sputorum	1	0.40	Health
Haemophilus sputorum	1.95	2.79	Health
Haemophilus sputorum	2.47	2.22	Health
Haemophilus sputorum	2.19	1.63	Health
Haemophilus sputorum	1.78	0.60	Health
Haemophilus sputorum	2.77	0.32	Health
Haemophilus sputorum	1.86	1.06	Health
Haemophilus sputorum	1	0.61	Health
Porphyromonas gingivalis	1	0.53	FluA/FluB
Porphyromonas gingivalis	1.04	8.68	FluB
Porphyromonas gingivalis	1	0.21	FluA
Porphyromonas gingivalis	1.14	8.29	FluB
Porphyromonas gingivalis	1.06	1.42	FluA/FluB
Porphyromonas gingivalis	1.43	0.66	FluA
Porphyromonas gingivalis	1.37	1.84	FluA
Porphyromonas gingivalis	1.28	0.28	FluA
Porphyromonas gingivalis	1.28	1.93	FluA/FluB
Porphyromonas gingivalis	1.11	4.60	FluA
Porphyromonas gingivalis	1.13	1.09	FluB
Porphyromonas gingivalis	1.17	4.63	FluA
Porphyromonas gingivalis	1.28	1.38	FluA/FluB
Porphyromonas gingivalis	1.18	0.58	FluA
Porphyromonas gingivalis	1.2	4.49	FluA/FluB
Porphyromonas gingivalis	1.15	0.45	FluB
Porphyromonas gingivalis	1.3	1.62	FluB
Porphyromonas gingivalis	1.19	0.94	Health
Porphyromonas gingivalis	1.13	1.31	Health
Porphyromonas gingivalis	1.04	4.97	Health
Porphyromonas gingivalis	1.24	6.23	Health
Porphyromonas gingivalis	1.11	1.69	Health
Porphyromonas gingivalis	1.1	0.73	Health
Porphyromonas gingivalis	1.1	2.41	Health
Treponema denticola	1.27	0.30	FluB
Treponema denticola	1.14	0.44	FluB
Treponema denticola	1.48	0.48	FluA
Treponema denticola	1.15	0.59	FluB
Treponema denticola	1.4	1.23	FluA/FluB
Treponema denticola	1.47	0.31	FluA/FluB

Treponema denticola	1.27	0.72	FluA
Treponema denticola	1.23	0.41	FluB
Treponema denticola	1.41	0.25	FluA/FluB
Treponema denticola	1.28	0.32	FluA
Treponema denticola	1.22	0.28	FluA
Treponema denticola	1.19	0.50	FluA/FluB
Treponema denticola	1.25	0.41	FluB
Treponema denticola	1.13	0.28	Health
Treponema denticola	1.3	0.22	Health
Treponema denticola	1.21	0.39	Health
Treponema denticola	1.21	0.22	Health
Treponema denticola	1.09	0.48	Health
Treponema medium	1.5	0.25	FluA/FluB
Treponema medium	1.64	0.41	FluB
Treponema medium	1.36	0.41	FluB
Treponema medium	1.36	0.54	FluA
Treponema medium	1.37	0.55	FluA
Treponema medium	1.4	1.03	FluB
Treponema medium	1.42	1.00	FluA/FluB
Treponema medium	1.58	0.40	FluA
Treponema medium	1.49	0.73	FluA/FluB
Treponema medium	1.49	0.71	FluA
Treponema medium	1.28	0.44	FluB
Treponema medium	1.27	0.71	FluA/FluB
Treponema medium	1.48	0.33	FluA
Treponema medium	1.72	0.24	FluA/FluB
Treponema medium	1.56	0.53	FluA
Treponema medium	1.57	0.23	FluA/FluB
Treponema medium	1.42	0.70	FluA/FluB
Treponema medium	1.57	0.39	FluB
Treponema medium	1.42	0.31	FluB
Treponema medium	1.46	0.45	FluB
Treponema medium	1.22	0.32	Health
Treponema medium	1.28	0.29	Health
Treponema medium	1.35	1.14	Health
Treponema medium	1.44	0.48	Health
Treponema medium	1.38	0.42	Health
Treponema medium	1.18	0.45	Health

Table S6. The Spearman interaction of all pathways, ARGs, and pathogens.

**Objects (A & B means A interacts with B)**	***R* value**
Porphyromonas gingivalis & PWY-5088: L-glutamate degradation VIII	-0.583
Haemophilus influenzae & PWY-6305: putrescine biosynthesis IV	-0.656
Haemophilus influenzae & P161-PWY: acetylene degradation	-0.592
Haemophilus influenzae & PWY-7431: aromatic biogenic amine degradation	-0.567
Haemophilus influenzae & PWY0-1298: super-pathway of pyrimidine deoxyribonucleosides degradation	-0.514
Treponema medium & PWY-5005: biotin biosynthesis II	0.509
Treponema medium & PWY-6147: 6-hydroxymethyl-dihydropterin diphosphate biosynthesis I	0.575
Treponema medium & PWY-5897: superpathway of menaquinol-11 biosynthesis	0.611
Treponema medium & PWY-5898: superpathway of menaquinol-12 biosynthesis	0.611
Treponema medium & PWY-5899: superpathway of menaquinol-13 biosynthesis	0.611
Treponema medium & PWY-7282: 4-amino-2-methyl-5-phosphomethylpyrimidine biosynthesis	0.624
Treponema medium & THISYNARA-PWY: superpathway of thiamin diphosphate biosynthesis III	0.649
Treponema denticola & P122-PWY: heterolactic fermentation	-0.613
Treponema denticola & PWY-6269: adenosylcobalamin salvage from cobinamide II	0.537
Treponema medium & PWY-5837: 1,4-dihydroxy-2-naphthoate biosynthesis I	0.658
Treponema medium & PWY-5791: 1,4-dihydroxy-2-naphthoate biosynthesis II	0.658
Treponema denticola & P185-PWY: formaldehyde assimilation III	0.555
Treponema denticola & SALVADEHYPOX-PWY: adenosine nucleotides degradation II	0.580
Treponema denticola & PWY-6353: purine nucleotides degradation II	0.655
Treponema medium & gb|AGV10830_1|ARO_3002647|*APH(3_)-IIIa*	0.601
Haemophilus sputorum & PWY-7197: pyrimidine deoxyribonucleotide phosphorylation	0.519
Haemophilus sputorum & PWY-5861: superpathway of demethylmenaquinol-8 biosynthesis	0.520
Haemophilus sputorum & PWY-5838: superpathway of menaquinol-8 biosynthesis I	0.528
Haemophilus sputorum & PWY-6168: flavin biosynthesis III	0.531
Haemophilus sputorum & PWY-1269: CMP-3-deoxy-D-manno-octulosonate biosynthesis I	0.542
Haemophilus sputorum & GLYCOL-GLYOXDEG-PWY: superpathway of glycol metabolism and degradation	0.566
Treponema denticola & gb|BAE77933_1|ARO_3000518|*CRP*	-0.565
Treponema denticola & gb|YP_007503840_1|ARO_3000165|*tet(A)*	-0.552
