# Supplementary material for: Unique microbial landscape in the human oropharynx during different types of acute respiratory tract infections
Source: Microbiome. 2023 Jul 24;11:157. doi: 10.1186/s40168-023-01597-9 (PMC10364384; doi:10.1186/s40168-023-01597-9)
Supplement: Supplementary file 3 — Additional file 2: Figure S1. Multiple qPCR standard curves for FluA, FluB, RSV, and HR. Figure S2. Reads count and proportions of microbiota in sequencing profiles. (A) Microbial reads count in 16S rRNA gene sequencing data. (B) The proportions of microbiota in 16S rRNA gene sequencing data. (C) Microbial and human reads count in metagenomic sequencing data. Figure S3. The variation of microbial composition between virus-positive and negative groups. PCoA based on Bray–Curtis distances was analyzed, and PERMANOVA was used to evaluate the variation. Figure S4. The differential genera, pathway, and ARGs between health and FluA, FluB or FluAB group. (A) Compared to healthy controls, the functional pathways (MetaCyc database) with significant differences in virus infection were calculated. The number and color represent the fold change relative to the control group. Blank blocks indicate no significant difference. (B) The number of antibiotic resistance genes (ARGs) based on metagenomic data was used to evaluate the diversity of ARGs, and the variation between the infected and healthy groups was counted by Kruskal–Wallis test and Wilcoxon test with subsequent Bonferroni correction. (C) Significantly different resistance genes compared to healthy controls were shown. (D) LEfSe was used to calculate the different genera between healthand FluA, FluB, orFluAB participants, respectively. Figure S5. The relative abundance difference of potential pathogens between infected and healthy subjects. (A) Nine pathogenic species (in the blue circle) were identified in the oropharyngeal samples based on metagenomics sequencing analysis. (B) Kruskal-Wallis test and Wilcoxon test was used to calculate the relative abundance difference of nine pathogens between the infected and the healthy subjects. Figure S6. The growth rate of potential pathogens (in the blue circle) was detected based on metagenomics sequencing analysis. [file 40168_2023_1597_MOESM2_ESM.pdf]

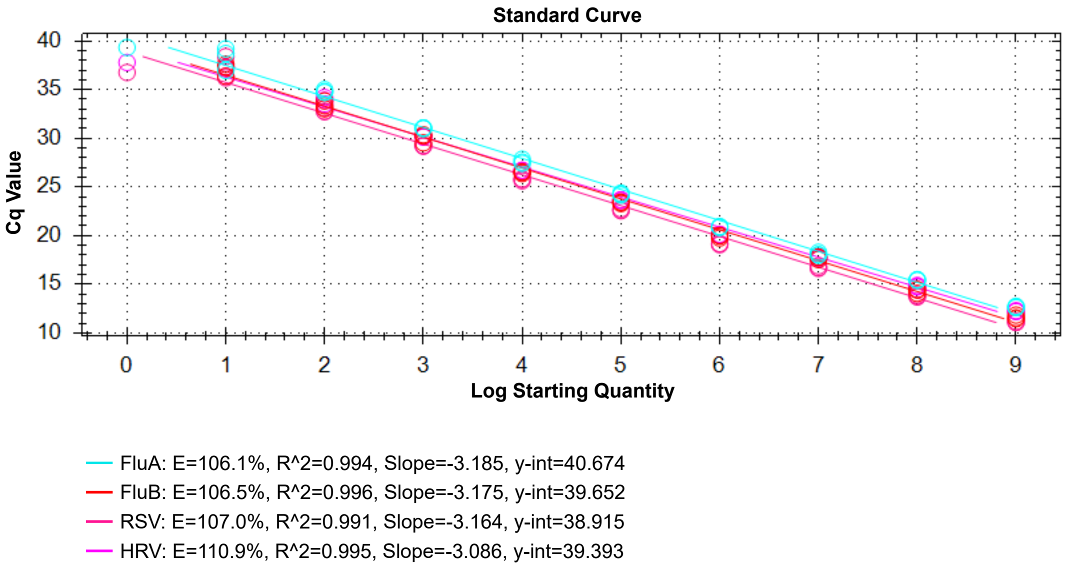

Figure S1. Multiple qPCR standard curves for FluA, FluB, RSV, and HRV.

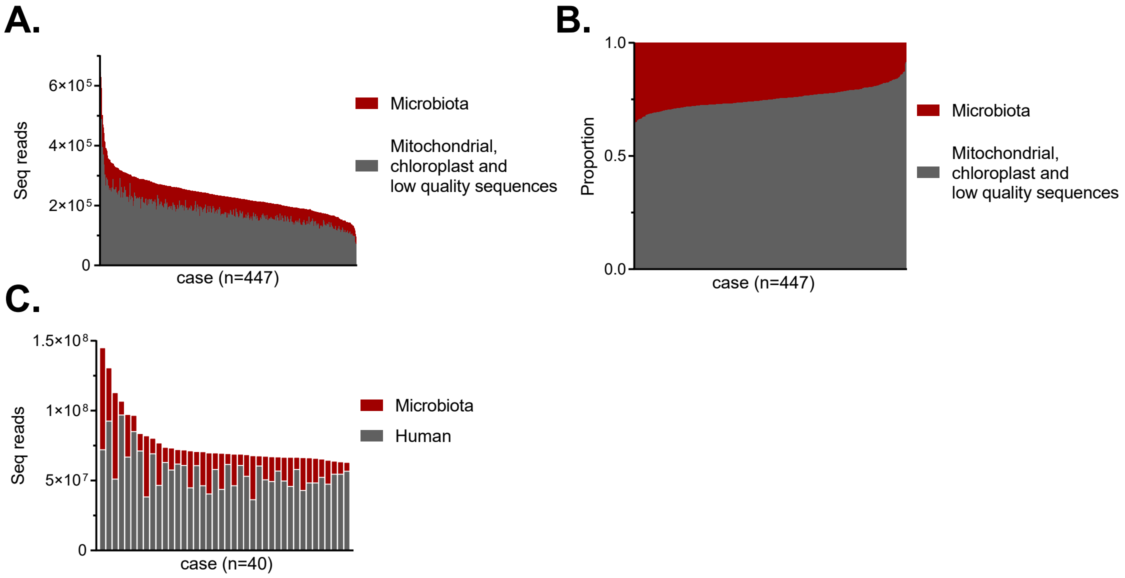

Figure S2. Reads count and proportions of microbiota in sequencing profiles. (A) Microbial reads count in 16S rRNA gene sequencing data. (B) The proportions of microbiota in 16S rRNA gene sequencing data. (C) Microbial and human reads count in metagenomic sequencing data.

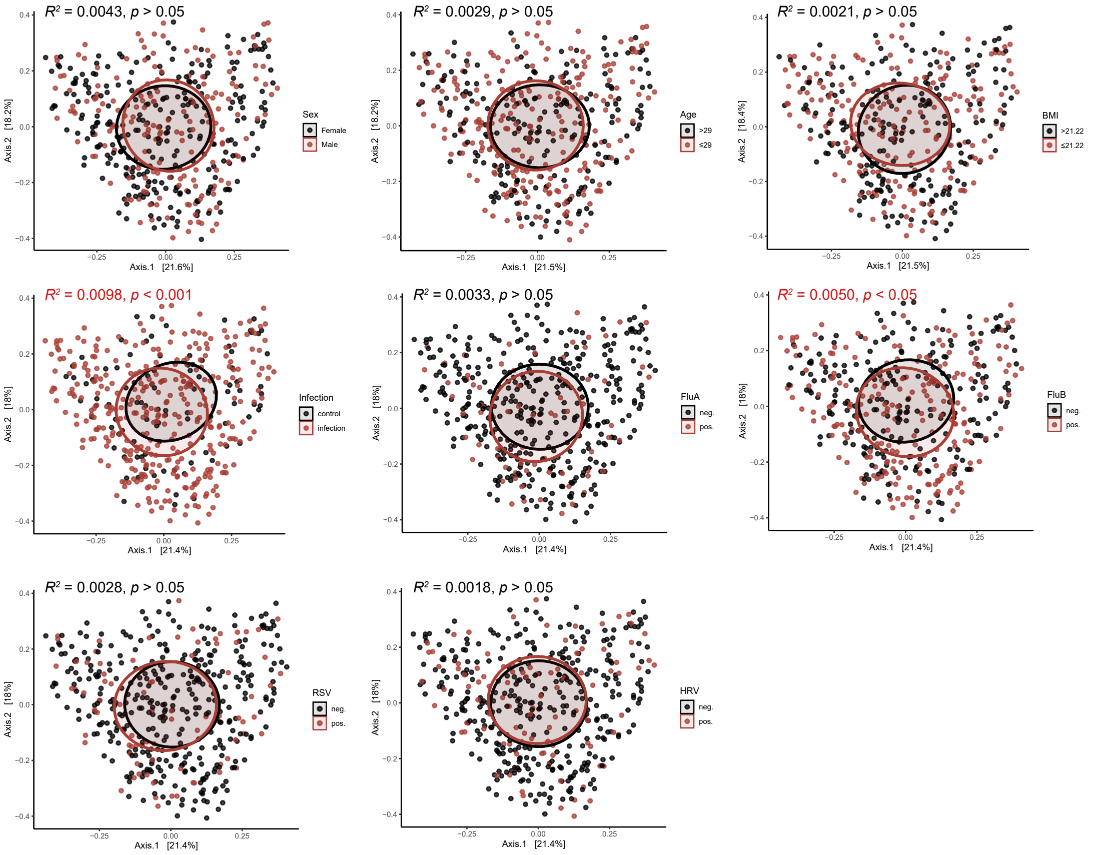

Figure S3. The variation of microbial composition between virus-positive and negative groups. PCoA based on Bray-Curtis distances was analyzed, and PERMANOVA was used to evaluate the variation

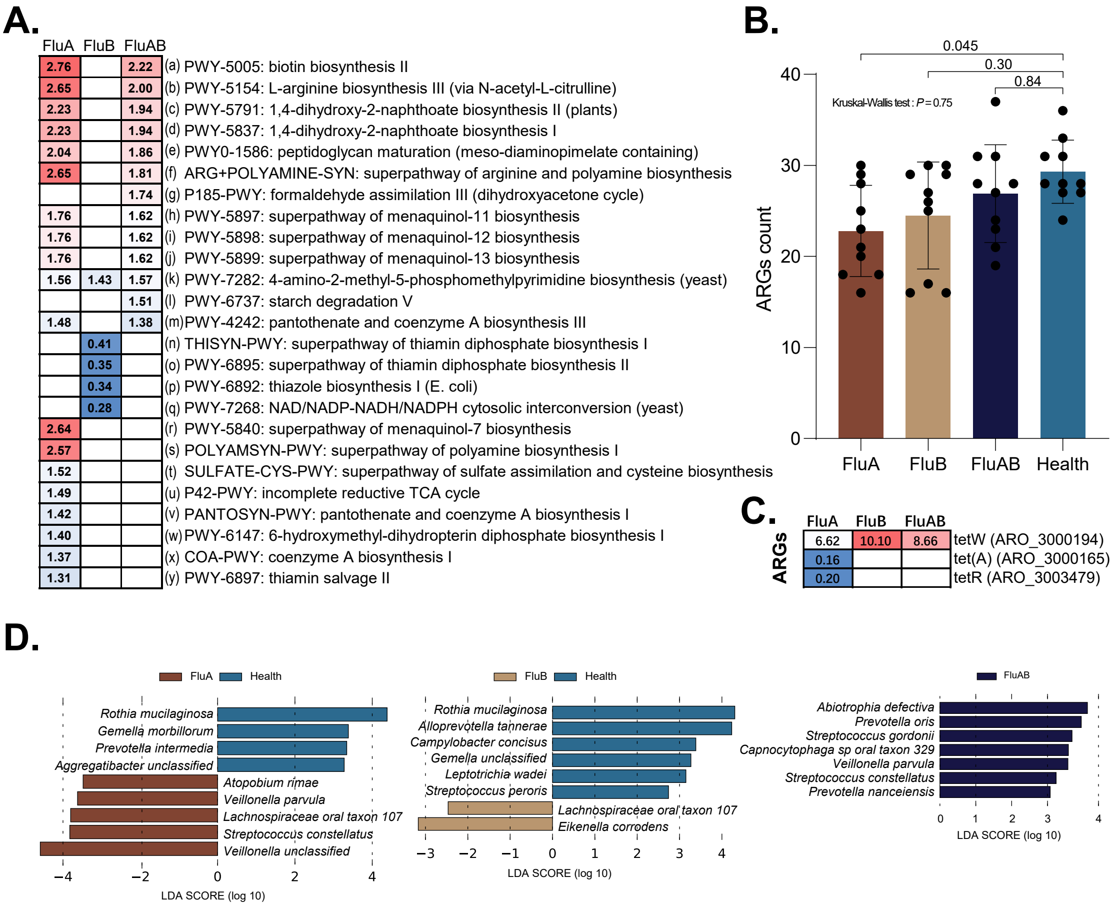

Figure S4. The differential genera, pathway, and ARGs between health and FluA, FluB or FluAB group. (A) Compared to healthy controls, the functional pathways (MetaCyc database) with significant differences in virus infection were calculated. The number and color represent the fold change relative to the control group. Blank blocks indicate no significant difference. (B) The number of antibiotic resistance genes (ARGs) based on metagenomic data was used to evaluate the diversity of ARGs, and the variation between the infected and healthy groups was counted by Kruskal-Wallis test and Wilcoxon test with subsequent Bonferroni correction. (C) Significantly different resistance genes compared to healthy controls were shown. (D) LEfSe was used to calculate the different genera between healthand FluA, FluB, orFluAB participants, respectively.

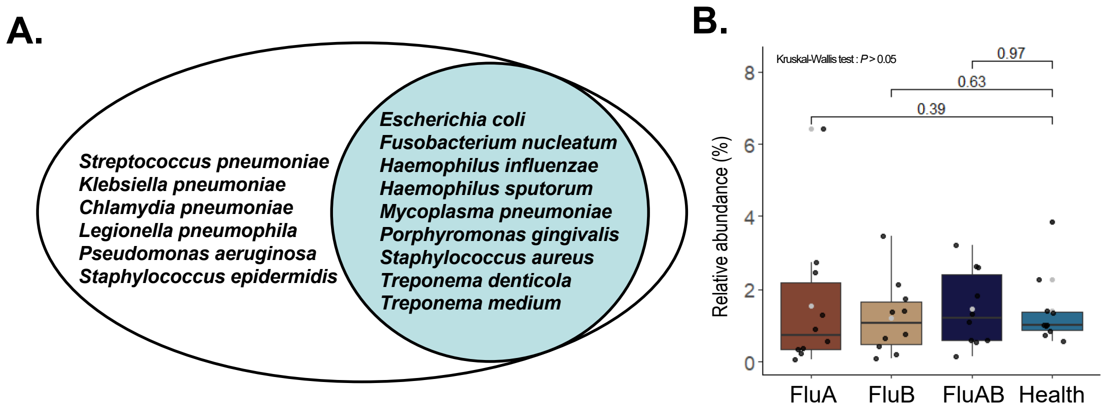

Figure S5. The relative abundance difference of potential pathogens between infected and healthy subjects. (A) Nine pathogenic species (in the blue circle) were identified in the oropharyngeal samples based on metagenomics sequencing analysis. (B) Kruskal-Wallis test and Wilcoxon test was used to calculate the relative abundance difference of nine pathogens between the infected and the healthy subjects.

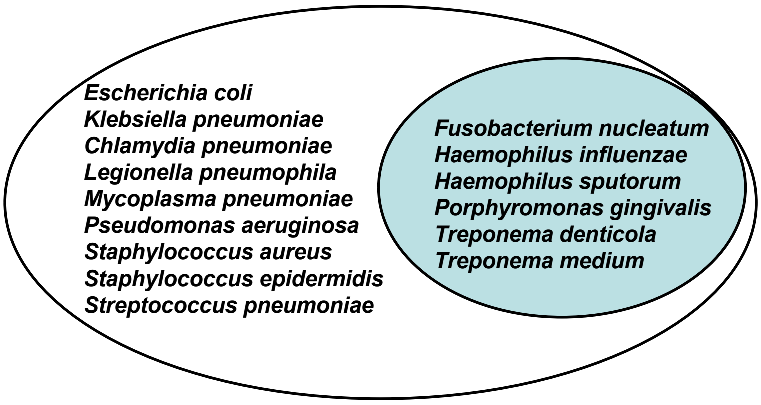

Figure S6. The growth rate of potential pathogens (in the blue circle) was detected based on metagenomics sequencing analysis.
